# Supplementary material for: Physicians’ emotion awareness and emotion regulation training during medical education: a systematic scoping review protocol
Source: BMJ Open. 2024 May 15;14(5):e080643. doi: 10.1136/bmjopen-2023-080643 (PMC11097880; doi:10.1136/bmjopen-2023-080643)
Supplement: Author's manuscript [file bmjopen-2023-080643.draft_revisions.pdf]

BMJ Open is committed to open peer review. As part of this commitment we make the peer review history of every article we publish publicly available.

When an article is published we post the peer reviewers' comments and the authors' responses online. We also post the versions of the paper that were used during peer review. These are the versions that the peer review comments apply to.

The versions of the paper that follow are the versions that were submitted during the peer review process. They are not the versions of record or the final published versions. They should not be cited or distributed as the published version of this manuscript.

BMJ Open is an open access journal and the full, final, typeset and author-corrected version of record of the manuscript is available on our site with no access controls, subscription charges or pay-per-view fees (http://bmjopen.bmj.com).

If you have any questions on BMJ Open's open peer review process please email info.bmjopen@bmj.com

BMJ Open

Physicians' emotions and emotion skills training during medical education: a systematic scoping review protocol

Journal:	*BMJ Open*
Manuscript ID	bmjopen-2023-080643
Article Type:	Protocol
Date Submitted by the Author:	06-Oct-2023
Complete List of Authors:	Lange, Anna; University of Bern, Institute of Social Preventive Medicine (ISPM); University of Bern, Graduate School of Health Sciences<br>Bonvin, Raphaël ; University of Fribourg, Department of Community Health<br>Guttormsen, Sissel; Universität Bern Institut für Medizinische Lehre<br>Zambrano Ramos, Sofia ; University of Bern Institute of Social and Preventive Medicine, Institute of Social Preventive Medicine (ISPM)
Keywords:	MEDICAL EDUCATION & TRAINING, Systematic Review, Self Care, Physicians

SCHOLARONE™
Manuscripts

Physicians' emotions and emotion skills training during medical education: a systematic scoping review protocol

Author list: Anna Lange (0000-0002-8150-3712) [1,4], Raphaël Bonvin (0000-0001-9322-2494) [2], Sissel Guttormsen Schär (0000-0001-9932-1872) [3], Sofia C. Zambrano (0000-0002-0918-9722) [1]

Affiliations

[1] Institute of Social Preventive Medicine (ISPM), University of Bern, Mittelstrasse 43, 3012 Bern, Switzerland

[2] Department of Community Health, University of Fribourg, Rte des Arsenaux 41 1700 Fribourg, Switzerland

[3] Institute for Medical Education (IML), University of Bern, Mittelstrasse 43, 3012 Bern, Switzerland

[4] Graduate School of Health Sciences, University of Bern, Mittelstrasse 43, 3012 Bern, Switzerland

Anna Lange, PhD Candidate.
Raphaël Bonvin, Director of Medical Education Unit.
Sissel Guttormsen Schär, Director of Institute for Medical Education.
Sofia C. Zambrano, Assistant professor.

Corresponding author:
Anna Lange, MSc Psychology
anna.lange@unibe.ch
Mittelstrasse 43, 3012 Bern, Institute of Social Preventive Medicine (ISPM), University of Bern, Bern, Switzerland

Word count manuscript: 2'213

Key words (6): Emotion skills training; Medical education; Self-directed emotions; Scoping review protocol; medical students; Medical Curriculum

For peer review only

Abstract

Introduction

The objective of this systematic scoping review is to identify what approaches have been implemented in medical education programs to teach medical students the skills to identify and manage emotions that may be elicited in them during physician-patient interactions and in the clinical environment. Emotions of all involved in the clinical encounter are central to the process of clinical care. However, a gap remains addressing and teaching medical students about recognising and dealing with their own emotions.

Methods and analysis

This scoping review will follow the updated JBI methodology guidance for the conduct and reporting of systematic scoping reviews, and the Preferred Reporting Items for Systematic reviews and Meta-Analyses extension for Scoping Reviews (PRISMA-ScR). A search strategy was developed and applied to five databases. Terms used included medical education, medical curriculum, medical students, emotion (regulation), psychological wellbeing and mental health. Additionally, a grey literature and reference list search will be conducted. Two independent reviewers will first screen titles and abstracts followed by a second, full-text screening phase. Publications to be included will contain information and data about teaching approaches such as lectures, and other teaching material on physicians' own emotions, and self-directed emotion skills training in medical education.

Ethics and dissemination

This study will review existing literature on emotion and emotion skills training in medical education, and as a systematic scoping review does not require ethical approval. The results of this scoping review will be submitted for publication to relevant peer-reviewed journals and will be used to inform the development and implementation of training programs and research studies aimed at preparing medical students to identify and manage their own emotions in the clinical environment.

For peer review only

Strengths and limitations of this study

- This protocol describes a systematic scoping review process, which will be the first to map out existing approaches to teach medical students about their own emotions and self-directed emotion skills training.
- For standardization and to meet the quality of other systematic scoping reviews, this scoping review will be conducted in accordance with the JBI methodology for scoping reviews and the Preferred Reporting Items for Systematic reviews and Meta-Analyses extension for Scoping Reviews (PRISMA-ScR) Checklist.
- To make the screening phase more systematic and the results more reliable, two independent reviewers will perform the first (title and abstract) and second (full-text) screening phases.
- The search strategy is expansive to provide best insight into the field of interest.
- To standardize the extraction of results during the data analysis phase, an extraction tool to chart the data has been developed and will be piloted prior to being used.

For peer review only

Introduction

A connection between clinicians and patients is central to clinical care (Brown-Johnson et al., 2019). Both, clinicians and patients have emotions, show emotions and will interpret each other's emotions (Roter et al., 2006; Schwartz et al., 2021). Despite their centrality, the clinicians' own emotions have largely remained unaddressed in medical education and practice (Holt Siropaides et al., 2021), and have often been deemed as unprofessional (Kerasidou & Horn, 2016; Sung et al., 2009; Zambrano et al., 2012), leaving clinicians with few skills to identify and manage their emotions. Within emotion management skills, a distinction can be made between 'other-directed' and 'self-directed' emotion skills. 'Other-directed' emotion skills are intended to improve the awareness, understanding and/or management of emotions/emotional states in others, while self-directed emotion skills are aimed to improve awareness, understanding and/or management of emotions/emotional states in the self, such as inward reflection or awareness of own emotions (Satterfield & Hughes, 2007). Since some emotion skills can be allocated to both (e.g. making soothing statements), a clearer distinction can be made according to the intended aim and focus of the emotion skill. To date, the majority of emotion skills training in medical education has been focused on emotions directed to the other, for example, emphasizing the development of empathy or compassion skills towards patients (Satterfield & Hughes, 2007).

Nevertheless, some competency frameworks of reference for medical training include and expect some self-directed emotion awareness and other related skills. The CanMEDS framework was firstly developed in 1990 and serves as a base for many derived frameworks worldwide, foremost describing the competencies which physicians have to develop during their education (Royal College of Physicians and Surgeons of Canada, n.d.). In the CanMEDS, in their role as Professionals, physicians are expected to have an "applied capacity for self-regulation, including the assessment and monitoring of one's thoughts, behaviours, emotions, and attention for optimal performance and well-being", as well as to have a "mindful and reflective approach to practice", and "resilience for sustainable practice" (Royal College of Physicians and Surgeons of Canada, n.d.). For the training of medical

For peer review only

students, Switzerland recently introduced the PROFILES framework, which is based on a combination of competencies adapted from the CanMEDS framework and Entrustable Professional Activities (EPAs) (Sohrmann et al., 2020; Swiss Working Group for PROFILES Implementation (SWGPI), n.d.). Here, physician-led regulation and maintenance of the physician's own health, recognizing excessive stress or recognizing personal illness, as well as being aware of own limits, and seeking supervision, are some of the expected skills that medical students should learn to display as professionals.

Despite these expectations from training, there seems to remain a gap in medical education in addressing and teaching physicians and medical students the skills to identify and manage their own emotions triggered by human interactions within the clinical care environment (Doulougeri et al., 2016; Linda Hadorn, 2020; Shapiro, 2011; Ungar et al., 2022), and to recognize the impact they may have on clinical care or on the clinicians' wellbeing.

Significant high rates of poor mental health including burnout, emotional exhaustion, depression, anxiety, and suicidal ideation are well documented in medical students and in practicing physicians (Almutairi et al., 2022; Devi, 2011; Ernst et al., 2021; Tugnoli et al., 2022). However, the number of preventative interventions and programs remain sparse and scattered in clinical practice and medical education. Some articles point out factors, such as chronically high workloads or lack of work-life balance which can help explain medical students' and physicians' decreased wellbeing (IsHak et al., 2013; West et al., 2018). Other studies point to the important role that emotional blunting, numbness or frequent suppression of own emotions, due to the lack of emotion skills, have on the poor mental health of medical students and physicians (Ardenghi et al., 2021; Dobkin & Hutchinson, 2013; Doulougeri et al., 2016; Linda Hadorn, 2020; Shapiro, 2011, 2012).

This scoping review will focus on identifying approaches to teaching medical students about the role of their own emotions in patient care along with the methods employed for self-directed emotion skills training. To classify the different teaching methods, we will employ the structure developed by Mitchell et al. (2023), which includes lectures, workshops, and other emotion skills training initiatives. For the development of future recommendations and interventions, it is necessary

For peer review only - http://bmjopen.bmj.com/site/about/guidelines.xhtml

to obtain a comprehensive understanding of current teaching approaches to 'self-directed' emotion skills training being implemented in medical education. For this purpose, a scoping review is justified and appropriate. A preliminary search of MEDLINE, the Cochrane Database of Systematic Reviews and *JBI Evidence Synthesis* was conducted and no current or underway systematic reviews or scoping reviews on the topic of medical students' management of own emotions or self-directed emotion skills training were identified. One related work identified has a focus on 'other-directed' emotion skills training in medical education (Satterfield & Hughes, 2007), and therefore the need for an equivalent work completing the picture of self-directed teaching approaches remains. This review will provide invaluable understandings that will facilitate the development of future training initiatives.

Objectives

The objective of this scoping review is to identify what approaches have been implemented in medical education programs to teach medical students the skills to identify and manage emotions that they may experience during physician-patient interactions and in the clinical environment in general.

Research questions

1. What specific teaching approaches, including lectures and training programs have been used in medical education to address the emotions physicians experience in the clinical environment?
2. With what teaching methods do specific lectures or related teaching formats cover elements related to physicians' 'self-directed' emotion skills training during medical education?
3. At what stage during medical education do specific lectures or related teaching formats cover elements related to training physicians to recognize and manage their own emotions?
4. Which specific aspects of physicians' own emotions, and which specific emotions, are addressed in 'self-directed' emotion skills training initiatives in medical education?

For peer review only - http://bmjopen.bmj.com/site/about/guidelines.xhtml

Methods and analysis

Eligibility Criteria

The PCC (Participants, Concept, Context) eligibility criteria of the JBI framework has been used (Peters et al., 2022).

Participants/Population

Studies to be included must report on students during medical school training, excluding those who have obtained the professional degree to practice as physicians and who may have progressed to residency, specialty training, or who may have already started to practice as junior doctors. Studies reporting on students from other related fields (e.g. nursing, dentistry, or physiotherapy, etc.) are to be excluded.

Concept

Literature to be included reports on teaching elements of medical programs, workshops implemented, or related implementation formats of content aimed at addressing emotions physicians may experience during clinical encounters or in the clinical environment, as well as 'self-directed' emotion skills training. Literature is to be excluded if only descriptive of mental health of medical students, only addressing needed changes on the topic of training of physicians' emotions without implementing teaching elements, or reporting on related concepts without elements of physicians' self-directed emotion skills (e.g., other-directed emotion teaching, relaxation training, etc.).

Context

To identify teaching approaches, studies to be included must report on the medical teaching environment, either about online or campus-based programs, seminars, interventions, or related teaching formats. International studies from all countries and languages are to be included, no limit back to a certain year will be set to include the literature.

For peer review only

Types of evidence sources

In this review, we will include experimental and quasi-experimental study designs including randomized controlled trials, non-randomized controlled trials, and before and after studies. Exploratory study designs, such as qualitative or mixed method studies or related study designs will also be included. In addition, studies of any design describing the interventions but not the specific outcomes will also be included. We will exclude books, editorials, comments, posters, and opinion pieces.

Support

Two research librarians/medical information specialists of the department of the ISPM of the University of Bern supported the development of the search strategy with their expertise. Further consultations with the research librarians are planned throughout the review process.

Registration

This scoping review protocol is registered in Figshare (10.6084/m9.figshare.23642208). Any adjustments or modifications made throughout the analysis process will be documented and stated in the final scoping review publication. An updated version of the scoping protocol will be available at Figshare.

Search strategy

This scoping review will be conducted in accordance with the JBI methodology for scoping reviews (Peters et al., 2022) and the Preferred Reporting Items for Systematic reviews and Meta-Analyses extension for Scoping Reviews (PRISMA-ScR) Checklist (Tricco et al., 2018). This scoping review protocol follows PRISMA-P guidelines (Moher et al. 2015) by employing the items recommended for

For peer review only

scoping review protocols by Peters et al. (2022). The protocol was developed using the template from the JBI Scoping Review Methodology Group (JBI Scoping Review Methodology Group, n.d.).

After some initial meetings to define the focus of the research strategy and the keywords, the key concepts were chosen, and the final search strategy was peer reviewed with the librarians before it was run. The final key concepts and the search strategy for one database can be found in Table 1. For the literature search, a sensitive approach was chosen (Peters et al., 2020). The following databases were searched: Medline (Ovid), PsychInfo (Ovid), ERIC (Ovid), CINAHL (EBSCOhost) and Web of Science – Social Sciences Citation Index SSCI (Clarivate, from inception to May 15st, 2023). No Language restrictions were applied. A total of 6726 articles were identified. The search strategy combined terms related to the population (medical students, trainees, or residents), exposure (medical education, teaching methods (undergraduate), curriculum) and outcomes (emotion regulation, psychological wellbeing, mental health). In databases where a thesaurus was available, articles were searched by thesaurus terms, in other databases by free text-terms in title and abstract. Duplicate records were removed by using Deduklick (Borissov et al., 2022), a fully automated deduplication algorithm. The full search strategies of all databases will be included in the appendix of the final scoping review publication.

Additionally, a grey literature search will be conducted, screening relevant literature references found through included full-texts and through hand searches. This search will include books and other sources such as MedEdPortal.org and similar resources. Experts in the field will also be asked for additional publications. An adapted PRISMA 2020 flow diagram describing the individual data sources and its output will be included in the scoping review publication (Page et al., 2021).

Table 1: Key concepts for one full search strategy at Medline ALL (via Ovid)

**1) Medical education, teaching methods (undergraduates), curriculum**
(education, medical/ or education, medical, undergraduate/ or exp Curriculum/ or exp Educational Technology/ or (((medical or clinical OR hidden OR informal) adj2 (curricul* or training* or educat* or school or teaching or undergraduate*)) or Entrustable Professional Activit* or "Situations as Starting Point*" or "problem-based" or "project-based*" or ((teaching or training) adj3 (method* or type* or form* or strateg* or intervention* or technique* or setting*)) or academic training* or pedagog* or paedagog* or handbook* or "how to teach" or ((new or innovat* or

For peer review only - http://bmjopen.bmj.com/site/about/guidelines.xhtml

classroom* or peer* or practice-based or bed-side or bedside or community or interactive* or creativ* or experimental* or precision or reflective) adj3 (teach*)) or "innovat* method*" or "diagnostic teaching" or ((small or large or discussion* or project*) adj1 group*) or "meet the expert*" or tutorial* or "hands on" or "psychomotor skills training*" or "case method*" or "case study method*" or "feedback session*" or debat* or quiz* or brainstorm* or lecture* or presentation* or "hot topic*" or webinar* or seminar* or symposi* or "case-based stud*" or "panel discussion*" or video* or movie* or film* or cinema* or "TED talk*" or "knowledge test*" or "facilitat* question* answer*" or "poster presentation*" or simulation* or "role-play*" or interview* or "patient simulat*" or "flipped classroom*" or forum* or game* or gaming or gamification or "skill demonstration*" or "skills demonstration*" or "mobile teaching app*" or "social media" or "scenario based" or "ward-round*" or "grand round*" or "practice exercise*" or "shadow expert*" or "inquiry-based" or "programmed instruct*" or "study assignment teach*" or "audiovisual instruction*" or "direct instruction*" or "individualized instruction*" or "individualised instruction*" or microteaching or "micro-teaching" or "multimedia instruction*" or scaffolding or "web based instruction*" or tutoring or workshop* or "virtual classroom*" or "group instruction*" or "formative assessment*" or debrief* or "information and communication technolog*").ab,ti,kf)
**2) Medical students**
(Students, Medical/ OR (((medical or healthcare or health-care or medicine) adj1 (student* or trainee* or resident*))).ab,ti)
**3) Emotion (regulation), psychological wellbeing, mental health**
(Emotions/ OR Mental Health/ OR (emotion or emotions or feelings or "emotional intelligen*" or emotionalit* or "emotional competenc*" or self-aware* or self-regulat* or (emotion* adj3 regulat*) or ((emotion* or intens*) adj2 reaction*) or "intens* feeling*" or self-reflect* or "reflective practice*" or self-care* or mindfulness or "self compassion*" or "coping strateg*" or psychological wellbeing or psychological well-being or mental health).ab,ti)
**4) Limits (no conference abstracts, letters, etc.)**
NOT (letter or news or comment or editorial or congress).pt.
**Translation of search strategy into Medline ALL (via Ovid)**
Segment PPEZ (Ovid MEDLINE® Epub Ahead of Print, In-Process & Other Non-Indexed Citations, Ovid MEDLINE® Daily, Ovid MEDLINE and Versions® 1946 to Present with Daily & Weekly Update)<br><br>(education, medical/ or education, medical, undergraduate/ or exp Curriculum/ or exp Educational Technology/ or (((medical or clinical OR hidden OR informal) adj2 (curricul* or training* or educat* or school or teaching or undergraduate*)) or Entrustable Professional Activit* or "Situations as Starting Point*" or "problem-based" or "project-based*" or ((teaching or training) adj3 (method* or type* or form* or strateg* or intervention* or technique* or setting*)) or academic training* or pedagog* or paedagog* or handbook* or "how to teach" or ((new or innovat* or classroom* or peer* or practice-based or bed-side or bedside or community or interactive* or creativ* or experimental* or precision or reflective) adj3 (teach*)) or "innovat* method*" or "diagnostic teaching" or ((small or large or discussion* or project*) adj1 group*) or "meet the expert*" or tutorial* or "hands on" or "psychomotor skills training*" or "case method*" or "case study method*" or "feedback session*" or debat* or quiz* or brainstorm* or lecture* or presentation* or "hot topic*" or webinar* or seminar* or symposi* or "case-based stud*" or "panel discussion*" or video* or movie* or film* or cinema* or "TED talk*" or "knowledge test*" or "facilitat* question* answer*" or "poster presentation*" or simulation* or "role-play*" or interview* or "patient simulat*" or "flipped classroom*" or forum* or game* or gaming or gamification or "skill demonstration*" or "skills demonstration*" or "mobile teaching app*" or "social media" or "scenario based" or "ward-round*" or "grand round*" or "practice exercise*" or "shadow expert*" or "inquiry-based" or "programmed instruct*" or "study assignment teach*" or "audiovisual instruction*" or "direct instruction*" or "individualized instruction*" or "individualised instruction*" or microteaching or "micro-teaching" or "multimedia instruction*" or scaffolding or "web based instruction*" or tutoring or workshop* or "virtual classroom*" or "group instruction*" or "formative assessment*" or debrief* or "information and communication technolog*").ab,ti,kf) AND (Students, Medical/ or (((medical or healthcare or health-care or medicine) adj1 (student* or trainee* or resident*))).ab,ti) AND (Emotions/ or Mental Health/ or (emotion or emotions or feelings or "emotional intelligen*" or emotionalit* or "emotional competenc*" or self-aware* or self-regulat* or (emotion* adj3 regulat*) or ((emotion* or intens*) adj2 reaction*) or "intens* feeling*" or self-reflect* or "reflective practice*" or self-care* or mindfulness or "self compassion*" or "coping strateg*" or psychological wellbeing or psychological well-being or mental health).ab,ti) NOT (letter or news or comment or editorial or congress).pt

For peer review only - http://bmjopen.bmj.com/site/about/guidelines.xhtml

Data Extraction

For documenting and managing the search results, the Rayyan software will be used (Ouzzani et al., 2016), as well as Endnote (The EndNote Team, 2013). In a first step, identified literature will be screened by the first author and by a second reviewer independently at a title and abstract level. This will be followed by a full-text check of the literature that complied with the inclusion criteria during the first screening process. The full-text screening will also be performed by the two reviewers independently. For piloting purposes of the accuracy and sensitivity of the inclusion and exclusion criteria, 20% of the papers will be screened by the first and last author of this protocol. In case of disagreement at any of the screening phases, discussions will be held between the two screeners, and when needed, a third reviewer will be involved to reach an agreement.

Of the full texts selected in the second screening phase, data will be extracted for analyses. To chart the data, an extraction tool (Table 2) has been developed and will be piloted to test its feasibility. After the piloting, possible adaptations of the extraction tool will be made, and the final completed extraction tool will be published in the scoping review. As the studies to be included may contain interventions, the extraction tool includes a combination of applicable items from the TIDieR (Template for Intervention Description and Replication) and the PAGER (Patterns, Advances, Gaps, Evidence for practice, Research recommendations) frameworks, as well as additional variables added by the authors. Data will be extracted to a cloud-based spreadsheet, to allow for collaboration, tracking of changes, and secure storage. Once data extraction has been completed, the datasheet will be imported for analyses into NVivo (Lumivero, 2023).

Table 2: Extraction Tool. The rows of this table represent the columns of the extraction tool to be used.

Source of evidence (citation)
Database found in
Year publication
Country, City
Name Medical School

For peer review only - http://bmjopen.bmj.com/site/about/guidelines.xhtml

Year study conducted	
Participants	*N*
	No M, No F, MD age
Lecture/Course/Training/Intervention<br><br>**TIDieR Framework and additional items developed by the authors**	*Title*
	rationale/theory/goal of elements essential to the intervention
	Nature of intervention
	Main competencies addressed/ main competencies taught
	Materials: physical or informational used for delivery; access where
	Procedures: each of procedures/activities and/or processes used in intervention (including enabling or support activities)
	Who provided: expertise, background, training for each intervention provider
	modes of delivery, provided individually or in group
	type(s) of location(s) for intervention (including infrastructure, etc.)
	No of interventions, over what period of time, number of sessions, schedule, duration, intensity/dose
	Who received? (year of study, which students)
	Mandatory (M) or Elective Training (M vs. ET)
	tailoring planned? If yes: what, why, when, how
	Modifications during course of study? If yes: what, why, when, how
	was intervention fidelity assessed? If yes: how, by whom, which strategies used to maintain or improve
	if fidelity assessed: describe extent to which intervention was delivered as panned
**PAGER Framework**	*Pattern*
	Advances
	Gaps
	Evidence for practice
	Research reccomendations

Data Analyses and Presentation

The analysis of the data will be narrative and descriptive, presenting the results in forms of diagrams and tables and describing the literature included in terms of conceptual categories such as intervention type, duration of intervention, timing of the intervention across the curriculum, key findings, research gaps, as well as related categories relevant to the study questions.

Discussion

This scoping review will provide an overview of the approaches used in medical education to teach physicians on identifying and managing the emotions they may experience during physician-patient interactions or in the clinical environment, including 'self-directed' emotion skills training. The review could help identify common themes, formats, and methods used to teach medical students about how to manage their own emotions, the effectiveness of these initiatives, and identify gaps in the curriculum of medical students in that very field of training and education for their future as physicians. Ultimately, the results of this scoping review could provide recommendations to inform the future implementation of training programs in medical education to address physicians' own emotions in the clinical environment.

Declarations

Patient and Public involvement

There is no direct patient or public engagement planned for this project. This is a systematic scoping review of the existing peer reviewed literature on the topic of interest. It is possible, however, that some of the literature included for data analyses might have had patient engagement or public involvement to some degree.

Ethics and dissemination

This study will review existing literature on emotion and emotion skills training in medical education, and as a systematic scoping review does not require ethical approval. The results of this scoping review will be submitted for publication to relevant peer-reviewed journals and will be used to inform the

For peer review only

development and implementation of training programs and research studies aimed at preparing medical students to identify and manage their own emotions in the clinical environment.

Acknowledgements

The authors thank the librarian Beatrice Minder from the University Library of Bern for her support and help with designing the search strategies and Doris Kopp-Heim from the University Library of Bern for the peer-review of the searches using PRESS (Peer Review of Electronic Search Strategies: 2015 Guideline) (Mcgowan et al., 2016). The authors would also like to thank Lina Murer, master's student in Psychology, for supporting this project and for being the second independent screener of the literature screening process. This scoping review is to contribute towards the PhD degree of the first author (AL).

Funding

This scoping review is being funded by the Swiss National Science Foundation (SNSF) via an Eccellenza Professorial Fellowship grant to the last author (SCZ) (grant number PCEFP1_194177). The funder has had no role in the development of this protocol and will not be involved in any of the scientific steps required to complete the scoping review.

Author contributions

AL and SCZ conceived the idea of the study and developed the initial design of the scoping review. AL, RB, SGS, and SCZ contributed to the methodological design of the review. AL and SCZ drafted the manuscript. All authors critically revised the manuscript for important intellectual content. SCZ is the guarantor of the systematic scoping review.

Conflicts of interest disclosures

The authors do not have any conflicts of interest to report.

For peer review only

References

Almutairi, H., Alsubaiei, A., Abduljawad, S., Alshatti, A., Fekih-Romdhane, F., Husni, M., & Jahrami, H. (2022). Prevalence of burnout in medical students: A systematic review and meta-analysis. *International Journal of Social Psychiatry*, *68*(6), 1157-1170. https://doi.org/10.1177/00207640221106691

Ardenghi, S., Russo, S., Bani, M., Rampoldi, G., & Strepparava, M. G. (2021). The role of difficulties in emotion regulation in predicting empathy and patient-centeredness in pre-clinical medical students: a cross-sectional study. *Psychology, Health & Medicine*, 1-15. https://doi.org/10.1080/13548506.2021.2001549

Borissov, N., Haas, Q., Minder, B., Kopp-Heim, D., von Gernler, M., Janka, H., Teodoro, D., & Amini, P. (2022). Reducing systematic review burden using Deduklick: a novel, automated, reliable, and explainable deduplication algorithm to foster medical research. *Systematic Reviews*, *11*(1), 172. https://doi.org/10.1186/s13643-022-02045-9

Brown-Johnson, C., Schwartz, R., Maitra, A., Haverfield, M. C., Tierney, A., Shaw, J. G., Zionts, D. L., Safaeinili, N., Thadaney Israni, S., Verghese, A., & Zulman, D. M. (2019). What is clinician presence? A qualitative interview study comparing physician and non-physician insights about practices of human connection. *BMJ Open*, *9*(11), e030831. https://doi.org/10.1136/bmjopen-2019-030831

Devi, S. (2011). Doctors in distress. *The Lancet*, *377*(9764), 454-455. https://doi.org/https://dx.doi.org/10.1016/S0140-6736%2811%2960145-1

Dobkin, P. L., & Hutchinson, T. A. (2013). Teaching mindfulness in medical school: where are we now and where are we going? *Med Educ*, *47*(8), 768-779. https://doi.org/10.1111/medu.12200

Doulougeri, K., Panagopoulou, E., & Montgomery, A. (2016). (How) do medical students regulate their emotions? *BMC Medical Education*, *16*(1). https://doi.org/10.1186/s12909-016-0832-9

Ernst, J., Jordan, K.-D., Weilenmann, S., Sazpinar, O., Gehrke, S., Paolercio, F., Petry, H., Pfaltz, M. C., Méan, M., Aebischer, O., Gachoud, D., Morina, N., Von Känel, R., & Spiller, T. R. (2021). Burnout, depression and anxiety among Swiss medical students – A network analysis. *Journal of Psychiatric Research*, *143*, 196-201. https://doi.org/10.1016/j.jpsychires.2021.09.017

Holt Siropaides, C., Howell, M., & Chou, C. L. (2021). Teaching about Emotions in Healthcare. In R. Schwartz, J. A. Hall, & L. G. Osterberg (Eds.), *Emotion in the Clinical Encounter* (pp. 1-9). McGraw Hill.

IsHak, W., Nikravesh, R., Lederer, S., Perry, R., Ogunyemi, D., & Bernstein, C. (2013). Burnout in medical students: a systematic review. 242–245.

JBI Scoping Review Methodology Group. (n.d.). *JBI*. Retrieved 03.10.2023 from www.jbi.global/scoping-review-network/resources

Kerasidou, A., & Horn, R. (2016). Making space for empathy: supporting doctors in the emotional labour of clinical care. *Bmc Medical Ethics*, *17*(1), 8. https://doi.org/10.1186/s12910-016-0091-7

Linda Hadorn, B. W., Carlos B. Quinto. (2020). Nachholbedarf in Sachen Ärztegesundheit. *SCHWEIZERISCHE ÄRZTEZEITUNG*, *38*(101), 1171-1172. https://doi.org/10.4414/saez.2020.19213

Lumivero. (2023). *NVivo*. In (Version Version 14) QSR International Pty Ltd. 2018.

For peer review only

Mcgowan, J., Sampson, M., Salzwedel, D. M., Cogo, E., Foerster, V., & Lefebvre, C. (2016). PRESS Peer Review of Electronic Search Strategies: 2015 Guideline Statement. *Journal of Clinical Epidemiology*, *75*, 40-46. https://doi.org/10.1016/j.jclinepi.2016.01.021

Mitchell, S., Sehlbach, C., Franssen, G., Janczukowicz, J., & Guttormsen, S. (2023). A taxonomy of teaching methods and their use in health professions education: A Scoping Review Protocol. *n.d.*

Ouzzani, M., Hammady, H., Fedorowicz, Z., & Elmagarmid, A. (2016). Rayyan—a web and mobile app for systematic reviews. *Systematic Reviews*, *5*(1). https://doi.org/10.1186/s13643-016-0384-4

Page, M. J., Mckenzie, J. E., Bossuyt, P. M., Boutron, I., Hoffmann, T. C., Mulrow, C. D., Shamseer, L., Tetzlaff, J. M., Akl, E. A., Brennan, S. E., Chou, R., Glanville, J., Grimshaw, J. M., Hróbjartsson, A., Lalu, M. M., Li, T., Loder, E. W., Mayo-Wilson, E., Mcdonald, S., . . . Moher, D. (2021). The PRISMA 2020 statement: an updated guideline for reporting systematic reviews. *BMJ*, n71. https://doi.org/10.1136/bmj.n71

Peters, M., Godfrey, C., McInerney, P., Khalil, H., Larsen, P., Marnie, C., Pollock, D., Tricco, A., & Munn, Z. (2022). Best practice guidance and reporting items for the development of scoping review protocols. *JBI Evidence Synthesis*, *Publish Ahead of Print*. https://doi.org/10.11124/JBIES-21-00242

Peters, M. D. J., Marnie, C., Tricco, A. C., Pollock, D., Munn, Z., Alexander, L., McInerney, P., Godfrey, C. M., & Khalil, H. (2020). Updated methodological guidance for the conduct of scoping reviews. *JBI Evidence Synthesis*, *18*(10), 2119-2126. https://doi.org/10.11124/jbies-20-00167

Roter, D. L., Frankel, R. M., Hall, J. A., & Sluyter, D. (2006). The Expression of Emotion Through Nonverbal Behavior in Medical Visits. *Journal of General Internal Medicine*, *21*(S1), S28-S34. https://doi.org/https://doi.org/10.1111/j.1525-1497.2006.00306.x

Royal College of Physicians and Surgeons of Canada. (n.d.). *CanMEDS Framework*. Retrieved 19.06.2023 from www.royalcollege.ca/rcsite/canmeds/canmeds-framework-e

Satterfield, J. M., & Hughes, E. (2007). Emotion skills training for medical students: a systematic review. *Medical education*, *41*(10), 935-941. https://doi.org/10.1111/j.1365-2923.2007.02835.x

Schwartz, R., Hall, J. A., & Osterberg, L. G. (2021). *Emotion in the Clinical Encounter*. McGraw-Hill Education. https://books.google.ch/books?id=TtE1zgEACAAJ

Shapiro, J. (2011). Perspective: Does medical education promote professional alexithymia? A call for attending to the emotions of patients and self in medical training. *Acad Med*, *86*(3), 326-332. https://doi.org/10.1097/ACM.0b013e3182088833

Shapiro, J. (2012). The feeling physician: educating the emotions in medical training. *European Journal for Person Centered Healthcare*, *1*(2), 310-316.

Sohrmann, M., Berendonk, C., Nendaz, M., Bonvin, R., & Swiss Working Group For Profiles, I. (2020). Nationwide introduction of a new competency framework for undergraduate medical curricula: a collaborative approach. *Swiss Med Wkly*, *150*, w20201. https://doi.org/10.4414/smw.2020.20201

Sung, A. D., Collins, M. E., Smith, A. K., Sanders, A. M., Quinn, M. A., Block, S. D., & Arnold, R. M. (2009). Crying: experiences and attitudes of third-year medical students and interns. *Teaching & Learning in Medicine*, *21*(3), 180-187. https://doi.org/10.1080/10401330903014111

For peer review only - http://bmjopen.bmj.com/site/about/guidelines.xhtml

Swiss Working Group for PROFILES Implementation (SWGPI). (n.d.). *PROFILES*. Retrieved 19.06.2023 from www.profilesmed.ch

The EndNote Team. (2013). *EndNote*. In (Version EndNote 20) [64 bit]. Clarivate.

Tricco, A. C., Lillie, E., Zarin, W., O'Brien, K. K., Colquhoun, H., Levac, D., Moher, D., Peters, M. D. J., Horsley, T., Weeks, L., Hempel, S., Akl, E. A., Chang, C., Mcgowan, J., Stewart, L., Hartling, L., Aldcroft, A., Wilson, M. G., Garritty, C., . . . Straus, S. E. (2018). PRISMA Extension for Scoping Reviews (PRISMA-ScR): Checklist and Explanation. *Annals of Internal Medicine*, *169*(7), 467-473. https://doi.org/10.7326/m18-0850

Tugnoli, S., Casetta, I., Caracciolo, S., & Salviato, J. (2022). Parental bonding, depression, and suicidal ideation in medical students. *Frontiers in Psychology*, *13*, 877306. https://doi.org/https://dx.doi.org/10.3389/fpsyg.2022.877306

Ungar, P., Schindler, A. K., Polujanski, S., & Rotthoff, T. (2022). Online programs to strengthen the mental health of medical students: A systematic review of the literature. *Medical Education Online*, *27*(1), 2082909. https://doi.org/doi:https://dx.doi.org/10.1080/10872981.2022.2082909

West, C. P., Dyrbye, L. N., & Shanafelt, T. D. (2018). Physician burnout: contributors, consequences and solutions. *Journal of Internal Medicine*, *283*(6), 516-529. https://doi.org/10.1111/joim.12752

Zambrano, S. C., Chur-Hansen, A., & Crawford, B. (2012). On the emotional connection of medical specialists dealing with death and dying: a qualitative study of oncologists, surgeons, intensive care specialists and palliative medicine specialists. *BMJ Supportive & Palliative Care*, *2*(3), 270-275. https://doi.org/10.1136/bmjspcare-2012-000208

For peer review only - http://bmjopen.bmj.com/site/about/guidelines.xhtml

Physicians' emotions and emotion skills training during medical education: a systematic scoping review protocol
Lange et al., submitted 2023

Recommended items to report in a scoping review protocol (JBI Scoping Review Methodology Group, n.d.; Peters et al., 2022)

Section and topic	Item No	Checklist item	Page#
ADMINISTRATIVE INFORMATION			
Title: *Physicians' emotions and emotion skills training during medical education: a systematic scoping review protocol*			
Identification	1a	Identify the report as a protocol of a scoping review	1
Update	1b	If the protocol is for an update of a previous scoping review, identify as such	n.a.
Registration	2	If registered, provide the name of the registry (such as JBI) and registration number	8
Authors:			
Contact	3a	Provide name, institutional affiliation, email address of all protocol authors; provide physical mailing address of corresponding author	1
Contributions	3b	Describe contributions of protocol authors and identify the guarantor of the review	
Amendments	4	If the protocol represents an amendment of a previously completed or published protocol, identify as such and list changes; otherwise, state plan for documenting important protocol amendments	8
Support:			
Sources	5a	Indicate sources of financial or other support for the review	8, 14
Sponsor	5b	Provide name for the review funder and/or sponsor	14
Role of sponsor or funder	5c	Describe roles of funder(s), sponsor(s), and/or institution(s), if any, in developing the protocol	14
INTRODUCTION			
Rationale	6	Describe the rationale for the review in the context of what is already known (Note: Consider providing a rationale for the choice of conducting a scoping review as compared to other evidence synthesis approaches)	4 - 6
Objectives	7	Provide an explicit statement of the question(s) the review will address with reference to the inclusion/ exclusion criteria	6
METHODS			
Eligibility criteria	8	Specify the study characteristics (such as PICO, study design, setting, timeframe) and report characteristics (such as years considered, language, publication status) to be used as criteria for eligibility for the review	7 - 8
Information sources	9	Describe all intended information sources (such as electronic databases, contact with study authors, trial registers or other gray literature sources) with planned dates of coverage	8 - 9
Search strategy	10	Present draft of search strategy to be used for at least one electronic database, including planned limits, such that it could be repeated	9 - 10
Study records:			
Data management	11a	Describe the mechanism(s) that will be used to manage records and data throughout the review	11
Selection process	11b	State the process that will be used for selecting studies (such as two independent reviewers) through each phase of the review (that is, screening, eligibility and inclusion)	7 - 8
Data collection process	11c	Describe planned method of extracting data from reports (such as piloting forms, done independently, in duplicate), any processes for obtaining and confirming data from investigators	10 - 12
Data items	12	List and define all variables for which data will be sought (such as PICO items, funding sources), any preplanned data assumptions and simplifications (Note: Scoping reviews may not use PICO and instead may use JBI's Population, Concept, and Context [PCC] or another approach to reporting eligibility criteria)	11 - 12

For peer review only

Physicians' emotions and emotion skills training during medical education: a systematic scoping review protocol
Lange et al., submitted 2023

Outcomes and prioritization	13	List and define all outcomes for which data will be sought, including prioritization of main and additional outcomes, with rationale (Note: Scoping reviews may not extract outcome data, so this can refer to whichever data items are extracted)	11-12
Risk of bias in individual studies	14	If this is to occur, describe anticipated methods for assessing risk of bias of individual studies, including whether this will be done at the outcome or study level, or both; state how this information will be used in data synthesis (Note: Scoping reviews typically do not include risk of bias assessment, but this information should be described if it will occur)	n.a.
Data synthesis	15a	Describe criteria under which study data will be presented (Note: Scoping reviews do not typically include quantitative synthesis of study data, but should still describe in advance how extracted data are anticipated to be presented in the resulting review)	12
	15b	Describe the planned approach to how extracted data will be presented (such as figures, tables, evidence gaps maps)	12
	15c	Describe any proposed additional analyses (such as thematic analyses) (Note: The JBI methodological guidance does not recommend undertaking thematic analysis as this synthesis of data should ideally occur following methodological appraisal of the included sources)	12
	15d	If quantitative synthesis is not appropriate, describe the type of summary planned	12
Meta-bias(es)	16	Specify any planned assessment of meta-bias(es) (such as publication bias across studies, selective reporting within studies) (Note: Scoping reviews typically do not include assessment of meta-bias[es], but this information should be described if it will occur)	n.a.
Confidence in cumulative evidence	17	Describe how the strength of the body of evidence will be assessed (such as GRADE)(GRADE for scoping reviews currently does not exist, and at this stage it is unclear if a variation on GRADE would be useful)	n.a.

References

JBI Scoping Review Methodology Group. (n.d.). *JBI*. Retrieved 03.10.2023 from www.jbi.global/scoping-review-network/resources

Peters, M., Godfrey, C., McInerney, P., Khalil, H., Larsen, P., Marnie, C., Pollock, D., Tricco, A., & Munn, Z. (2022). Best practice guidance and reporting items for the development of scoping review protocols. *JBI Evidence Synthesis*, *Publish Ahead of Print*. https://doi.org/10.11124/JBIES-21-00242

For peer review only

Physicians' emotions and emotion skills training during medical education: a systematic scoping review protocol

Journal:	*BMJ Open*
Manuscript ID	bmjopen-2023-080643.R1
Article Type:	Protocol
Date Submitted by the Author:	02-Feb-2024
Complete List of Authors:	Lange, Anna; University of Bern, Institute of Social Preventive Medicine (ISPM); University of Bern, Graduate School of Health Sciences<br>Bonvin, Raphaël ; University of Fribourg, Department of Community Health<br>Guttormsen, Sissel; Universität Bern Institut für Medizinische Lehre<br>Zambrano Ramos, Sofia ; University of Bern Institute of Social and Preventive Medicine, Institute of Social Preventive Medicine (ISPM)
**Primary Subject Heading**:	Medical education and training
Secondary Subject Heading:	Medical education and training
Keywords:	MEDICAL EDUCATION & TRAINING, Systematic Review, Self Care, Physicians

SCHOLARONE™
Manuscripts

Physicians' emotions and emotion skills training during medical education: a systematic scoping review protocol

Author list: Anna Lange (0000-0002-8150-3712) [1,4], Raphaël Bonvin (0000-0001-9322-2494) [2], Sissel Guttormsen Schär (0000-0001-9932-1872) [3], Sofia C. Zambrano (0000-0002-0918-9722) [1]

Affiliations

[1] Institute of Social Preventive Medicine (ISPM), University of Bern, Mittelstrasse 43, 3012 Bern, Switzerland

[2] Department of Community Health, University of Fribourg, Rte des Arsenaux 41 1700 Fribourg, Switzerland

[3] Institute for Medical Education (IML), University of Bern, Mittelstrasse 43, 3012 Bern, Switzerland

[4] Graduate School of Health Sciences, University of Bern, Mittelstrasse 43, 3012 Bern, Switzerland

Anna Lange, PhD Candidate.
Raphaël Bonvin, Director of Medical Education Unit.
Sissel Guttormsen Schär, Director of Institute for Medical Education.
Sofia C. Zambrano, Assistant professor.

Corresponding author:
Anna Lange, MSc Psychology
anna.lange@unibe.ch
Mittelstrasse 43, 3012 Bern, Institute of Social Preventive Medicine (ISPM), University of Bern, Bern, Switzerland

Word count manuscript: 2'259

Key words (6): Emotion skills training; Medical education; Self-directed emotions; Scoping review protocol; medical students; Medical Curriculum

For peer review only

Abstract

Introduction

The objective of this systematic scoping review is to identify what approaches have been implemented in medical education programs to teach medical students the skills to identify and manage emotions that may be elicited in them during physician-patient interactions and in the clinical environment. Emotions of all involved in the clinical encounter are central to the process of clinical care. However, a gap remains addressing and teaching medical students about recognising and dealing with their own emotions.

Methods and analysis

This scoping review will follow the updated JBI methodology guidance for the conduct and reporting of systematic scoping reviews, and the Preferred Reporting Items for Systematic reviews and Meta-Analyses extension for Scoping Reviews (PRISMA-ScR). A search strategy was developed and applied to five databases. Terms used included medical education, medical curriculum, medical students, emotion (regulation), psychological wellbeing and mental health. Additionally, a grey literature and reference list search will be conducted. Two independent reviewers will first screen titles and abstracts followed by a second, full-text screening phase. Publications to be included will contain information and data about teaching approaches such as lectures, and other teaching material on physicians' own emotions, and self-directed emotion skills training in medical education.

Ethics and dissemination

This study will review existing literature on emotion and emotion skills training in medical education, and as a systematic scoping review does not require ethical approval. The results of this scoping review will be submitted for publication to relevant peer-reviewed journals and will be used to inform the development and implementation of training programs and research studies aimed at preparing medical students to identify and manage their own emotions in the clinical environment.

For peer review only

For peer review only - http://bmjopen.bmj.com/site/about/guidelines.xhtml

Strengths and limitations of this study

- For standardization and to meet the quality of other systematic scoping reviews, this scoping review will be conducted in accordance with the JBI methodology for scoping reviews and the Preferred Reporting Items for Systematic reviews and Meta-Analyses extension for Scoping Reviews (PRISMA-ScR) Checklist.
- To make the screening phase more systematic and the results more reliable, two independent reviewers will perform the first (title and abstract) and second (full-text) screening phases.
- The search strategy is expansive to provide best insight into the field of interest.
- To standardize the extraction of results during the data analysis phase, an extraction tool to chart the data has been developed and will be piloted prior to being used.

For peer review only

For peer review only - http://bmjopen.bmj.com/site/about/guidelines.xhtml

Introduction

A connection between clinicians and patients is central to clinical care [1]. Both, clinicians and patients have emotions, show emotions and will interpret each other's emotions [2, 3]. Despite their centrality, the clinicians' own emotions have largely remained unaddressed in medical education and practice [4], and have often been deemed as unprofessional [5-7], leaving clinicians with few skills to identify and manage their emotions. Within emotion management skills, a distinction can be made between 'other-directed' and 'self-directed' emotion skills. 'Other-directed' emotion skills are intended to improve the awareness, understanding and/or management of emotions/emotional states in others, while self-directed emotion skills are aimed to improve awareness, understanding and/or management of emotions/emotional states in the self, such as inward reflection or awareness of own emotions [8]. Since some emotion skills can be allocated to both (e.g. making soothing statements), a clearer distinction can be made according to the intended aim and focus of the emotion skill. To date, the majority of emotion skills training in medical education has been focused on emotions directed to the other, for example, emphasizing the development of empathy or compassion skills towards patients [8].

Nevertheless, some competency frameworks of reference for medical training include and expect some self-directed emotion awareness and other related skills. The CanMEDS framework was firstly developed in 1990 and serves as a base for many derived frameworks worldwide, foremost describing the competencies which physicians have to develop during their education [9]. In the CanMEDS, in their role as Professionals, physicians are expected to have an "applied capacity for self-regulation, including the assessment and monitoring of one's thoughts, behaviours, emotions, and attention for optimal performance and well-being", as well as to have a "mindful and reflective approach to practice", and "resilience for sustainable practice" [9]. For the training of medical students, Switzerland recently introduced the PROFILES framework, which is based on a combination of competencies adapted from the CanMEDS framework and Entrustable Professional Activities (EPAs) [10, 11]. Here, physician-led regulation and maintenance of the physician's own health, recognizing

For peer review only

excessive stress or recognizing personal illness, as well as being aware of own limits, and seeking supervision, are some of the expected skills that medical students should learn to display as professionals.

Despite these expectations from training, there seems to remain a gap in medical education in addressing and teaching physicians and medical students the skills to identify and manage their own emotions triggered by human interactions within the clinical care environment [12-15], and to recognize the impact they may have on clinical care or on the clinicians' wellbeing.

Significant high rates of poor mental health including burnout, emotional exhaustion, depression, anxiety, and suicidal ideation are well documented in medical students and in practicing physicians [16-19]. However, the number of preventative interventions and programs remain sparse and scattered in clinical practice and medical education. Some articles point out factors, such as chronically high workloads or lack of work-life balance which can help explain medical students' and physicians' decreased wellbeing [20, 21]. Other studies point to the important role that emotional blunting, numbness or frequent suppression of own emotions, due to the lack of emotion skills, have on the poor mental health of medical students and physicians [12-14, 22-24].

This scoping review will focus on identifying approaches to teaching medical students about the role of their own emotions in patient care along with the methods employed for self-directed emotion skills training. To classify the different teaching methods, we will employ the structure developed by Mitchell et al. [25], which includes lectures, workshops, and other emotion skills training initiatives. For the development of future recommendations and interventions, it is necessary to obtain a comprehensive understanding of current teaching approaches to 'self-directed' emotion skills training being implemented in medical education. For this purpose, a scoping review is justified and appropriate. A preliminary search of MEDLINE, the Cochrane Database of Systematic Reviews and *JBI Evidence Synthesis* was conducted and no current or underway systematic reviews or scoping reviews on the topic of medical students' management of own emotions or self-directed emotion skills training were identified. One related work identified has a focus on 'other-directed' emotion skills training in

For peer review only

medical education [8], and therefore the need for an equivalent work completing the picture of self-directed teaching approaches remains. This review will provide invaluable understandings that will facilitate the development of future training initiatives.

Objectives

The objective of this scoping review is to identify what approaches have been implemented in medical education programs to teach medical students the skills to identify and manage emotions that they may experience during physician-patient interactions and in the clinical environment in general.

Research questions

1. What specific teaching approaches, including lectures and training programs have been used in medical education to address the emotions physicians experience in the clinical environment?
2. With what teaching methods do specific lectures or related teaching formats cover elements related to physicians' 'self-directed' emotion skills training during medical education?
3. At what stage during medical education do specific lectures or related teaching formats cover elements related to training physicians to recognize and manage their own emotions?
4. Which specific aspects of physicians' own emotions, and which specific emotions, are addressed in 'self-directed' emotion skills training initiatives in medical education?

Ethics and dissemination

This study will review existing literature on emotion and emotion skills training in medical education, and as a systematic scoping review does not require ethical approval. The results of this scoping review will be submitted for publication to relevant peer-reviewed journals and will be used to inform the development and implementation of training programs and research studies aimed at preparing medical students to identify and manage their own emotions in the clinical environment.

For peer review only - http://bmjopen.bmj.com/site/about/guidelines.xhtml

Methods and analysis

Eligibility Criteria

The PCC (Participants, Concept, Context) eligibility criteria of the JBI framework has been used [26].

Patient and Public involvement

There is no direct patient or public engagement planned for this project. This is a systematic scoping review of the existing peer reviewed literature on the topic of interest. It is possible, however, that some of the literature included for data analyses might have had patient engagement or public involvement to some degree.

Participants/Population

Studies to be included must report on students during medical school training, excluding those who have obtained the professional degree to practice as physicians and who may have progressed to residency, specialty training, or who may have already started to practice as junior doctors. Studies reporting on students from other related fields (e.g. nursing, dentistry, or physiotherapy, etc.) are to be excluded.

Concept

Literature to be included reports on teaching elements of medical programs, workshops implemented, or related implementation formats of content aimed at addressing emotions physicians may experience during clinical encounters or in the clinical environment, as well as 'self-directed' emotion skills training. Literature is to be excluded if only descriptive of mental health of medical students, only addressing needed changes on the topic of training of physicians' emotions without implementing teaching elements, or only reporting on related concepts without addressing elements of physicians'

For peer review only

self-directed emotion and emotion skills (e.g., other-directed emotion teaching, relaxation training, etc.).

Context

To identify teaching approaches, studies to be included must report on the medical teaching environment, either about online or campus-based programs, seminars, interventions, or related teaching formats. International studies from all countries and languages are to be included, no limit back to a certain year will be set to include the literature.

Types of evidence sources

In this review, we will include experimental and quasi-experimental study designs including randomized controlled trials, non-randomized controlled trials, and before and after studies. Exploratory study designs, such as qualitative or mixed method studies or related study designs will also be included. In addition, studies of any design describing the interventions but not the specific outcomes will also be included. We will exclude books, editorials, comments, posters, and opinion pieces.

Support

Two research librarians/medical information specialists of the department of the ISPM of the University of Bern supported the development of the search strategy with their expertise. Further consultations with the research librarians are planned throughout the review process.

Registration

This scoping review protocol is registered in Figshare (10.6084/m9.figshare.23642208). Any adjustments or modifications made throughout the analysis process will be documented and stated

For peer review only

For peer review only - http://bmjopen.bmj.com/site/about/guidelines.xhtml

in the final scoping review publication. An updated version of the scoping protocol will be available at Figshare.

Search strategy

This scoping review will be conducted in accordance with the JBI methodology for scoping reviews [26] and the Preferred Reporting Items for Systematic reviews and Meta-Analyses extension for Scoping Reviews (PRISMA-ScR) Checklist [27]. This scoping review protocol follows PRISMA-P guidelines (Moher et al. 2015) by employing the items recommended for scoping review protocols by Peters, Godfrey [26]. The protocol was developed using the template from the JBI Scoping Review Methodology Group [28].

After some initial meetings to define the focus of the research strategy and the keywords, the key concepts were chosen, and the final search strategy was peer reviewed with the librarians before it was run. The final key concepts and the search strategy for one database can be found in Table 1. For the literature search, a sensitive approach was chosen [29]. The following databases were searched: Medline (Ovid), PsychInfo (Ovid), ERIC (Ovid), CINAHL (EBSCOhost) and Web of Science – Social Sciences Citation Index SSCI (Clarivate, from inception to May 15st, 2023). No Language restrictions were applied. A total of 6726 articles were identified. The search strategy combined terms related to the population (medical students, trainees, or residents), exposure (medical education, teaching methods (undergraduate), curriculum) and outcomes (e.g. emotion regulation, psychological wellbeing, or mental health). Despite our interest in medical students before obtaining the professional degree to practice as physicians, we included the terms "trainees" and "residents" in the search strategy to ensure that studies reporting on mixed populations were not excluded from the outset. In databases where a thesaurus was available, articles were searched by thesaurus terms, in other databases by free text-terms in title and abstract. Duplicate records were removed by using Deduklick [30], a fully automated deduplication algorithm. The full search strategies of all databases will be included in the appendix of the final scoping review publication.

For peer review only - http://bmjopen.bmj.com/site/about/guidelines.xhtml

Additionally, a grey literature search will be conducted, screening relevant literature references found through included full-texts and through hand searches. This search will include books and other sources such as MedEdPortal.org and similar resources. Experts in the field will also be asked for additional publications. An adapted PRISMA 2020 flow diagram describing the individual data sources and its output will be included in the scoping review publication [31].

Table 1: Key concepts for one full search strategy at Medline ALL (via Ovid)

**1) Medical education, teaching methods (undergraduates), curriculum**
(education, medical/ or education, medical, undergraduate/ or exp Curriculum/ or exp Educational Technology/ or (((medical or clinical OR hidden OR informal) adj2 (curricul* or training* or educat* or school or teaching or undergraduate*)) or Entrustable Professional Activit* or "Situations as Starting Point*" or "problem-based" or "project-based*" or ((teaching or training) adj3 (method* or type* or form* or strateg* or intervention* or technique* or setting*)) or academic training* or pedagog* or paedagog* or handbook* or "how to teach" or ((new or innovat* or classroom* or peer* or practice-based or bed-side or bedside or community or interactive* or creativ* or experimental* or precision or reflective) adj3 (teach*)) or "innovat* method*" or "diagnostic teaching" or ((small or large or discussion* or project*) adj1 group*) or "meet the expert*" or tutorial* or "hands on" or "psychomotor skills training*" or "case method*" or "case study method*" or "feedback session*" or debat* or quiz* or brainstorm* or lecture* or presentation* or "hot topic*" or webinar* or seminar* or symposi* or "case-based stud*" or "panel discussion*" or video* or movie* or film* or cinema* or "TED talk*" or "knowledge test*" or "facilitat* question* answer*" or "poster presentation*" or simulation* or "role-play*" or interview* or "patient simulat*" or "flipped classroom*" or forum* or game* or gaming or gamification or "skill demonstration*" or "skills demonstration*" or "mobile teaching app*" or "social media" or "scenario based" or "ward-round*" or "grand round*" or "practice exercise*" or "shadow expert*" or "inquiry-based" or "programmed instruct*" or "study assignment teach*" or "audiovisual instruction*" or "direct instruction*" or "individualized instruction*" or "individualised instruction*" or microteaching or "micro-teaching" or "multimedia instruction*" or scaffolding or "web based instruction*" or tutoring or workshop* or "virtual classroom*" or "group instruction*" or "formative assessment*" or debrief* or "information and communication technolog*").ab,ti,kf)
**2) Medical students**
(Students, Medical/ OR (((medical or healthcare or health-care or medicine) adj1 (student* or trainee* or resident*))).ab,ti)
**3) Emotion (regulation), psychological wellbeing, mental health**
(Emotions/ OR Mental Health/ OR (emotion or emotions or feelings or "emotional intelligen*" or emotionalit* or "emotional competenc*" or self-aware* or self-regulat* or (emotion* adj3 regulat*) or ((emotion* or intens*) adj2 reaction*) or "intens* feeling*" or self-reflect* or "reflective practice*" or self-care* or mindfulness or "self compassion*" or "coping strateg*" or psychological wellbeing or psychological well-being or mental health).ab,ti)
**4) Limits (no conference abstracts, letters, etc.)**
NOT (letter or news or comment or editorial or congress).pt.
**Translation of search strategy into Medline ALL (via Ovid)**
Segment PPEZ (Ovid MEDLINE® Epub Ahead of Print, In-Process & Other Non-Indexed Citations, Ovid MEDLINE® Daily, Ovid MEDLINE and Versions® 1946 to Present with Daily & Weekly Update)<br><br>(education, medical/ or education, medical, undergraduate/ or exp Curriculum/ or exp Educational Technology/ or (((medical or clinical OR hidden OR informal) adj2 (curricul* or training* or educat* or school or teaching or

For peer review only

undergraduate*)) or Entrustable Professional Activit* or "Situations as Starting Point*" or "problem-based" or "project-based*" or ((teaching or training) adj3 (method* or type* or form* or strateg* or intervention* or technique* or setting*)) or academic training* or pedagog* or paedagog* or handbook* or "how to teach" or ((new or innovat* or classroom* or peer* or practice-based or bed-side or bedside or community or interactive* or creativ* or experimental* or precision or reflective) adj3 (teach*)) or "innovat* method*" or "diagnostic teaching" or ((small or large or discussion* or project*) adj1 group*) or "meet the expert*" or tutorial* or "hands on" or "psychomotor skills training*" or "case method*" or "case study method*" or "feedback session*" or debat* or quiz* or brainstorm* or lecture* or presentation* or "hot topic*" or webinar* or seminar* or symposi* or "case-based stud*" or "panel discussion*" or video* or movie* or film* or cinema* or "TED talk*" or "knowledge test*" or "facilitat* question* answer*" or "poster presentation*" or simulation* or "role-play*" or interview* or "patient simulat*" or "flipped classroom*" or forum* or game* or gaming or gamification or "skill demonstration*" or "skills demonstration*" or "mobile teaching app*" or "social media" or "scenario based" or "ward-round*" or "grand round*" or "practice exercise*" or "shadow expert*" or "inquiry-based" or "programmed instruct*" or "study assignment teach*" or "audiovisual instruction*" or "direct instruction*" or "individualized instruction*" or "individualised instruction*" or microteaching or "micro-teaching" or "multimedia instruction*" or scaffolding or "web based instruction*" or tutoring or workshop* or "virtual classroom*" or "group instruction*" or "formative assessment*" or debrief* or "information and communication technolog*").ab,ti,kf) AND (Students, Medical/ or (((medical or healthcare or health-care or medicine) adj1 (student* or trainee* or resident*))).ab,ti) AND (Emotions/ or Mental Health/ or (emotion or emotions or feelings or "emotional intelligen*" or emotionalit* or "emotional competenc*" or self-aware* or self-regulat* or (emotion* adj3 regulat*) or ((emotion* or intens*) adj2 reaction*) or "intens* feeling*" or self-reflect* or "reflective practice*" or self-care* or mindfulness or "self compassion*" or "coping strateg*" or psychological wellbeing or psychological well-being or mental health).ab,ti) NOT (letter or news or comment or editorial or congress).pt

Data Extraction

For documenting and managing the search results, the Rayyan software will be used [32], as well as Endnote [33]. In a first step, identified literature will be screened by the first author and by a second reviewer independently at a title and abstract level. This will be followed by a full-text check of the literature that complied with the inclusion criteria during the first screening process. The full-text screening will also be performed by the two reviewers independently. For piloting purposes of the accuracy and sensitivity of the inclusion and exclusion criteria, 20% of the papers will be screened by the first and last author of this protocol. In case of disagreement at any of the screening phases, discussions will be held between the two screeners, and when needed, a third reviewer will be involved to reach an agreement.

Of the full texts selected in the second screening phase, data will be extracted for analysis. To chart the data, an extraction tool (Table 2) has been developed and will be piloted to test its feasibility. After the piloting, possible adaptations of the extraction tool will be made, and the final completed extraction tool will be published in the scoping review. As the studies to be included may contain interventions, the extraction tool includes a combination of applicable items from the TIDieR (Template for Intervention Description and Replication) and the PAGER (Patterns, Advances, Gaps,

For peer review only - http://bmjopen.bmj.com/site/about/guidelines.xhtml

Evidence for practice, Research recommendations) frameworks, as well as additional variables added by the authors. Data will be extracted using Covidence, a web-based software for systematic reviews, to allow for collaboration, tracking of changes, and secure storage. Once data extraction has been completed, the datasheet will be imported for analyses into MAXQDA [34].

Table 2: Extraction Tool. The rows of this table represent the columns of the extraction tool to be used.

Study characteristics / Patterns	*Study DOI*
	Title of publication
	Year of publication
	Name(s) of author(s)
	Email address corresponding author
	Aim(s) of the research study
	Country in which study was conducted
	Name Medical School / Institution where teaching was held
	Year(s) in which study was conducted
	Short description of year(s)/ period in which study was conducted
	Type of study design
	Emotions topic
Participants	*Healthcare area*
	Participant numbers total included (table)
	Age participant numbers total included (table)
	Participation numbers (completed intervention) (table)
	Age participation numbers (completed intervention) (table)
	Method of recruitment of participants (selection of: phone, Mail, email, medical clinic, during lecture/course, not reported, other)
TIDieR Framework and additional items added by the authors / Advances	*Aim of intervention*
	Brief name of intervention
	Main competencies addressed in the intervention / main competencies taught
	Specific context of intervention
	Materials: physical or informational used for delivery
	Procedures: each of procedures / activities and/or processes used in intervention (including enabling or support activities)
	Who provided the intervention
	Modes of delivery
	Delivery provided individually or in group?

For peer review only - http://bmjopen.bmj.com/site/about/guidelines.xhtml

	(selection of: group, individually, other)
	type(s) of location(s) for intervention (including infrastructure, etc.)
	Number of interventions
	Number of sessions intervention 1 was delivered
	Number of sessions intervention 2 was delivered
	Number of sessions intervention 3 was delivered
	Number of sessions intervention 4 was delivered
	Period of time intervention 1 was delivered
	Period of time intervention 2 was delivered
	Period of time intervention 3 was delivered
	Period of time intervention 4 was delivered
	Schedule
	Duration of session for Intervention 1
	Duration of session for Intervention 2
	Duration of session for Intervention 3
	Duration of session for Intervention 4
	Who received? (year of study, which students?) (open text field)
	Who received the intervention (selection box)<br>*(selection of: medical students during non-clinical years, medical students during clinical years, mixed medical students and postgraduates, mixed medical students and undergraduates (undergraduate: not at medical school yet), unclear, other)*
	Mandatory or elective training?<br>*(selection of: mandatory, elective, not reported, other)*
	Tailoring planned?<br>*(selection of: yes, no, other)*
	tailoring planned?
	If yes: what, why, when, how (for each modification)
	Modifications to intervention during course of study?<br>*(yes, no, other)*
	If yes (which modifications during course of study)<br>*(what, why, when, how / per for each modification)*
Outcomes	*Outcomes measured*<br>*(yes, no, other)*
	Outcomes assessed
	(Significant) Results / Conclusions / Outcomes
	Was program evaluated?<br>*(yes, no, other)*
Remaining part of PAGER framework that was not yet covered by the previous items	*Gaps / Research recommendations*
	Evidence for practice
	Limitations of the study (designs) reported by the author(s) / Challenges in training that may explain results
Source of funding	*Details source of funding*

Data Analyses and Presentation

For peer review only - http://bmjopen.bmj.com/site/about/guidelines.xhtml

The analysis of the data will be narrative and descriptive, presenting the results in forms of diagrams and tables and describing the literature included in terms of conceptual categories such as intervention type, duration of intervention, timing of the intervention across the curriculum, key findings, research gaps, as well as related categories relevant to the study questions.

Discussion

This scoping review will provide an overview of the approaches used in medical education to teach physicians on identifying and managing the emotions they may experience during physician-patient interactions or in the clinical environment, including 'self-directed' emotion skills training. The review could help identify common themes, formats, and methods used to teach medical students about how to manage their own emotions, the effectiveness of these initiatives, and identify gaps in the curriculum of medical students in that very field of training and education for their future as physicians. Ultimately, the results of this scoping review could provide recommendations to inform the future implementation of training programs in medical education to address physicians' own emotions in the clinical environment.

Declarations

Acknowledgements

The authors thank the librarian Beatrice Minder from the University Library of Bern for her support and help with designing the search strategies and Doris Kopp-Heim from the University Library of Bern for the peer-review of the searches using PRESS (Peer Review of Electronic Search Strategies: 2015 Guideline) [35]. The authors would also like to thank Lina Murer, master's student in Psychology, for supporting this project and for being the second independent screener of the literature screening process. This scoping review is to contribute towards the PhD degree of the first author (AL).

Funding

For peer review only

This scoping review is being funded by the Swiss National Science Foundation (SNSF) via an Eccellenza Professorial Fellowship grant to the last author (SCZ) (grant number PCEFP1_194177). The funder has had no role in the development of this protocol and will not be involved in any of the scientific steps required to complete the scoping review.

Author contributions

AL and SCZ conceived the idea of the study and developed the initial design of the scoping review. AL, RB, SGS, and SCZ contributed to the methodological design of the review. AL and SCZ drafted the manuscript. All authors critically revised the manuscript for important intellectual content. SCZ is the guarantor of the systematic scoping review.

Conflicts of interest disclosures

The authors do not have any conflicts of interest to report.

For peer review only

References

1. Brown-Johnson, C., et al., *What is clinician presence? A qualitative interview study comparing physician and non-physician insights about practices of human connection.* BMJ Open, 2019. **9**(11): p. e030831.
2. Schwartz, R., J.A. Hall, and L.G. Osterberg, *Emotion in the Clinical Encounter*. 2021: McGraw-Hill Education.
3. Roter, D.L., et al., *The Expression of Emotion Through Nonverbal Behavior in Medical Visits.* Journal of General Internal Medicine, 2006. **21**(S1): p. S28-S34.
4. Holt Siropaides, C., M. Howell, and C.L. Chou, *Teaching about Emotions in Healthcare*, in *Emotion in the Clinical Encounter*, R. Schwartz, J.A. Hall, and L.G. Osterberg, Editors. 2021, McGraw Hill. p. 1-9.
5. Sung, A.D., et al., *Crying: experiences and attitudes of third-year medical students and interns.* Teaching & Learning in Medicine, 2009. **21**(3): p. 180-187.
6. Zambrano, S.C., A. Chur-Hansen, and B. Crawford, *On the emotional connection of medical specialists dealing with death and dying: a qualitative study of oncologists, surgeons, intensive care specialists and palliative medicine specialists.* BMJ Supportive & Palliative Care, 2012. **2**(3): p. 270-275.
7. Kerasidou, A. and R. Horn, *Making space for empathy: supporting doctors in the emotional labour of clinical care.* BMC Medical Ethics, 2016. **17**(1): p. 8.
8. Satterfield, J.M. and E. Hughes, *Emotion skills training for medical students: a systematic review.* Medical Education, 2007. **41**(10): p. 935-941.
9. Royal College of Physicians and Surgeons of Canada. *CanMEDS Framework*. n.d. [cited 2023 19.06.2023]; Available from: www.royalcollege.ca/rcsite/canmeds/canmeds-framework-e
.
10. Swiss Working Group for PROFILES Implementation (SWGPI). *PROFILES*. n.d. [cited 2023 19.06.2023]; Available from: www.profilesmed.ch.
11. Sohrmann, M., et al., *Nationwide introduction of a new competency framework for undergraduate medical curricula: a collaborative approach.* Swiss Med Wkly, 2020. **150**: p. w20201.
12. Doulougeri, K., E. Panagopoulou, and A. Montgomery, *(How) do medical students regulate their emotions?* BMC Medical Education, 2016. **16**(1).
13. Linda Hadorn, B.W., Carlos B. Quinto, *Nachholbedarf in Sachen Ärztegesundheit.* SCHWEIZERISCHE ÄRZTEZEITUNG, 2020. **38**(101): p. 1171-1172.
14. Shapiro, J., *Perspective: Does medical education promote professional alexithymia? A call for attending to the emotions of patients and self in medical training.* Acad Med, 2011. **86**(3): p. 326-32.
15. Ungar, P., et al., *Online programs to strengthen the mental health of medical students: A systematic review of the literature.* Medical Education Online, 2022. **27**(1): p. 2082909.
16. Almutairi, H., et al., *Prevalence of burnout in medical students: A systematic review and meta-analysis.* International Journal of Social Psychiatry, 2022. **68**(6): p. 1157-1170.
17. Devi, S., *Doctors in distress.* The Lancet, 2011. **377**(9764): p. 454-455.
18. Tugnoli, S., et al., *Parental bonding, depression, and suicidal ideation in medical students.* Frontiers in Psychology, 2022. **13**: p. 877306.

For peer review only

19. Ernst, J., et al., *Burnout, depression and anxiety among Swiss medical students – A network analysis.* Journal of Psychiatric Research, 2021. **143**: p. 196-201.
20. West, C.P., L.N. Dyrbye, and T.D. Shanafelt, *Physician burnout: contributors, consequences and solutions.* Journal of Internal Medicine, 2018. **283**(6): p. 516-529.
21. IsHak, W., et al., *Burnout in medical students: a systematic review.* 2013: p. 242–245.
22. Ardenghi, S., et al., *The role of difficulties in emotion regulation in predicting empathy and patient-centeredness in pre-clinical medical students: a cross-sectional study.* Psychology, Health & Medicine, 2021: p. 1-15.
23. Shapiro, J., *The feeling physician: educating the emotions in medical training.* European Journal for Person Centered Healthcare, 2012. **1**(2): p. 310-316.
24. Dobkin, P.L. and T.A. Hutchinson, *Teaching mindfulness in medical school: where are we now and where are we going?* Med Educ, 2013. **47**(8): p. 768-79.
25. Mitchell, S., et al., *Taxonomy of teaching methods and their use in health professions education: a scoping review protocol.* BMJ Open, 2024. **14**(1): p. e077282.
26. Peters, M., et al., *Best practice guidance and reporting items for the development of scoping review protocols.* JBI evidence synthesis, 2022. **Publish Ahead of Print**.
27. Tricco, A.C., et al., *PRISMA Extension for Scoping Reviews (PRISMA-ScR): Checklist and Explanation.* Annals of Internal Medicine, 2018. **169**(7): p. 467-473.
28. JBI Scoping Review Methodology Group. *JBI*. n.d. [cited 2023 03.10.2023]; Available from: www.jbi.global/scoping-review-network/resources.
29. Peters, M.D.J., et al., *Updated methodological guidance for the conduct of scoping reviews.* JBI Evidence Synthesis, 2020. **18**(10): p. 2119-2126.
30. Borissov, N., et al., *Reducing systematic review burden using Deduklick: a novel, automated, reliable, and explainable deduplication algorithm to foster medical research.* Systematic Reviews, 2022. **11**(1): p. 172.
31. Page, M.J., et al., *The PRISMA 2020 statement: an updated guideline for reporting systematic reviews.* BMJ, 2021: p. n71.
32. Ouzzani, M., et al., *Rayyan—a web and mobile app for systematic reviews.* Systematic Reviews, 2016. **5**(1).
33. The EndNote Team, *EndNote*. 2013, Clarivate: Philadelphia, PA.
34. VERBI Software, *MAXQDA 2024*. 2021, VERBI Software: Berlin, Germany.
35. Mcgowan, J., et al., *PRESS Peer Review of Electronic Search Strategies: 2015 Guideline Statement.* Journal of Clinical Epidemiology, 2016. **75**: p. 40-46.

For peer review only

Physicians' emotions and emotion skills training during medical education: a systematic scoping review protocol Lange et al., submitted 2023

Recommended items to report in a scoping review protocol (JBI Scoping Review Methodology Group, n.d.; Peters et al., 2022)

Section and topic	Item No	Checklist item	Page#
ADMINISTRATIVE INFORMATION			
Title: *Physicians' emotions and emotion skills training during medical education: a systematic scoping review protocol*			
Identification	1a	Identify the report as a protocol of a scoping review	1
Update	1b	If the protocol is for an update of a previous scoping review, identify as such	n.a.
Registration	2	If registered, provide the name of the registry (such as JBI) and registration number	8
Authors:			
Contact	3a	Provide name, institutional affiliation, email address of all protocol authors; provide physical mailing address of corresponding author	1
Contributions	3b	Describe contributions of protocol authors and identify the guarantor of the review	
Amendments	4	If the protocol represents an amendment of a previously completed or published protocol, identify as such and list changes; otherwise, state plan for documenting important protocol amendments	8
Support:			
Sources	5a	Indicate sources of financial or other support for the review	8, 14
Sponsor	5b	Provide name for the review funder and/or sponsor	14
Role of sponsor or funder	5c	Describe roles of funder(s), sponsor(s), and/or institution(s), if any, in developing the protocol	14
INTRODUCTION			
Rationale	6	Describe the rationale for the review in the context of what is already known (Note: Consider providing a rationale for the choice of conducting a scoping review as compared to other evidence synthesis approaches)	4 - 6
Objectives	7	Provide an explicit statement of the question(s) the review will address with reference to the inclusion/ exclusion criteria	6
METHODS			
Eligibility criteria	8	Specify the study characteristics (such as PICO, study design, setting, timeframe) and report characteristics (such as years considered, language, publication status) to be used as criteria for eligibility for the review	7 - 8
Information sources	9	Describe all intended information sources (such as electronic databases, contact with study authors, trial registers or other gray literature sources) with planned dates of coverage	8 - 9
Search strategy	10	Present draft of search strategy to be used for at least one electronic database, including planned limits, such that it could be repeated	9 - 10
Study records:			
Data management	11a	Describe the mechanism(s) that will be used to manage records and data throughout the review	11
Selection process	11b	State the process that will be used for selecting studies (such as two independent reviewers) through each phase of the review (that is, screening, eligibility and inclusion)	7 - 8
Data collection process	11c	Describe planned method of extracting data from reports (such as piloting forms, done independently, in duplicate), any processes for obtaining and confirming data from investigators	10 - 12
Data items	12	List and define all variables for which data will be sought (such as PICO items, funding sources), any preplanned data assumptions and simplifications (Note: Scoping reviews may not use PICO and instead may use JBI's Population, Concept, and Context [PCC] or another approach to reporting eligibility criteria)	11 - 12

For peer review only

Physicians' emotions and emotion skills training during medical education: a systematic scoping review protocol Lange et al., submitted 2023

Outcomes and prioritization	13	List and define all outcomes for which data will be sought, including prioritization of main and additional outcomes, with rationale (Note: Scoping reviews may not extract outcome data, so this can refer to whichever data items are extracted)	11-12
Risk of bias in individual studies	14	If this is to occur, describe anticipated methods for assessing risk of bias of individual studies, including whether this will be done at the outcome or study level, or both; state how this information will be used in data synthesis (Note: Scoping reviews typically do not include risk of bias assessment, but this information should be described if it will occur)	n.a.
Data synthesis	15a	Describe criteria under which study data will be presented (Note: Scoping reviews do not typically include quantitative synthesis of study data, but should still describe in advance how extracted data are anticipated to be presented in the resulting review)	12
	15b	Describe the planned approach to how extracted data will be presented (such as figures, tables, evidence gaps maps)	12
	15c	Describe any proposed additional analyses (such as thematic analyses) (Note: The JBI methodological guidance does not recommend undertaking thematic analysis as this synthesis of data should ideally occur following methodological appraisal of the included sources)	12
	15d	If quantitative synthesis is not appropriate, describe the type of summary planned	12
Meta-bias(es)	16	Specify any planned assessment of meta-bias(es) (such as publication bias across studies, selective reporting within studies) (Note: Scoping reviews typically do not include assessment of meta-bias[es], but this information should be described if it will occur)	n.a.
Confidence in cumulative evidence	17	Describe how the strength of the body of evidence will be assessed (such as GRADE)(GRADE for scoping reviews currently does not exist, and at this stage it is unclear if a variation on GRADE would be useful)	n.a.

References

JBI Scoping Review Methodology Group. (n.d.). *JBI*. Retrieved 03.10.2023 from www.jbi.global/scoping-review-network/resources

Peters, M., Godfrey, C., McInerney, P., Khalil, H., Larsen, P., Marnie, C., Pollock, D., Tricco, A., & Munn, Z. (2022). Best practice guidance and reporting items for the development of scoping review protocols. *JBI Evidence Synthesis*, *Publish Ahead of Print*. https://doi.org/10.11124/JBIES-21-00242

For peer review only

BMJ Open

Physicians' emotion awareness and emotion regulation training during medical education: a systematic scoping review protocol

Journal:	*BMJ Open*
Manuscript ID	bmjopen-2023-080643.R2
Article Type:	Protocol
Date Submitted by the Author:	07-Mar-2024

Complete List of Authors:	Lange, Anna; University of Bern, Institute of Social Preventive Medicine (ISPM); University of Bern, Graduate School of Health Sciences<br>Bonvin, Raphaël ; University of Fribourg, Department of Community Health<br>Guttormsen, Sissel; Universität Bern Institut für Medizinische Lehre<br>Zambrano Ramos, Sofia ; University of Bern Institute of Social and Preventive Medicine, Institute of Social Preventive Medicine (ISPM)

**Primary Subject Heading**:	Medical education and training
Secondary Subject Heading:	Medical education and training
Keywords:	MEDICAL EDUCATION & TRAINING, Systematic Review, Self Care, Physicians

SCHOLARONE™
Manuscripts

Physicians' emotion awareness and emotion regulation training during medical education: a systematic scoping review protocol

Author list: Anna Lange (0000-0002-8150-3712) [1,4], Raphaël Bonvin (0000-0001-9322-2494) [2], Sissel Guttormsen Schär (0000-0001-9932-1872) [3], Sofia C. Zambrano (0000-0002-0918-9722) [1]

Affiliations

[1] Institute of Social Preventive Medicine (ISPM), University of Bern, Mittelstrasse 43, 3012 Bern, Switzerland

[2] Department of Community Health, University of Fribourg, Rte des Arsenaux 41 1700 Fribourg, Switzerland

[3] Institute for Medical Education (IML), University of Bern, Mittelstrasse 43, 3012 Bern, Switzerland

[4] Graduate School of Health Sciences, University of Bern, Mittelstrasse 43, 3012 Bern, Switzerland

Anna Lange, PhD Candidate.
Raphaël Bonvin, Director of Medical Education Unit.
Sissel Guttormsen Schär, Director of Institute for Medical Education.
Sofia C. Zambrano, Assistant professor.

Corresponding author:
Anna Lange, MSc Psychology
anna.lange@unibe.ch
Mittelstrasse 43, 3012 Bern, Institute of Social Preventive Medicine (ISPM), University of Bern, Bern, Switzerland

Word count manuscript: 2'269

Key words (6): Emotion skills training; Medical education; Self-directed emotions; Scoping review protocol; medical students; Medical Curriculum

For peer review only

Abstract

Introduction

The objective of this systematic scoping review is to identify what approaches have been implemented in medical education programs to teach medical students the skills to identify and manage emotions that may be elicited in them during physician-patient interactions and in the clinical environment. Emotions of all involved in the clinical encounter are central to the process of clinical care. However, a gap remains addressing and teaching medical students about recognising and dealing with their own emotions.

Methods and analysis

This scoping review will follow the updated JBI methodology guidance for the conduct and reporting of systematic scoping reviews, and the Preferred Reporting Items for Systematic reviews and Meta-Analyses extension for Scoping Reviews (PRISMA-ScR). A search strategy was developed and applied to five databases. Terms used included medical education, medical curriculum, medical students, emotion (regulation), psychological wellbeing and mental health. Additionally, a grey literature and reference list search will be conducted. Two independent reviewers will first screen titles and abstracts followed by a second, full-text screening phase. Publications to be included will contain information and data about teaching approaches such as lectures, and other teaching material on physicians' emotion awareness and emotion regulation training in medical education.

Ethics and dissemination

This study will review existing literature on emotion awareness and emotion regulation training in medical education, and as a systematic scoping review does not require ethical approval. The results of this scoping review will be submitted for publication to relevant peer-reviewed journals and will be used to inform the development and implementation of training programs and research studies aimed at preparing medical students to identify and manage their own emotions in the clinical environment.

For peer review only

Strengths and limitations of this study

- For standardization and to meet the quality of other systematic scoping reviews, this scoping review will be conducted in accordance with the JBI methodology for scoping reviews and the Preferred Reporting Items for Systematic reviews and Meta-Analyses extension for Scoping Reviews (PRISMA-ScR) Checklist.
- To make the screening phase more systematic and the results more reliable, two independent reviewers will perform the first (title and abstract) and second (full-text) screening phases.
- The search strategy is expansive to provide best insight into the field of interest.
- To standardize the extraction of results during the data analysis phase, an extraction tool to chart the data has been developed and will be piloted prior to being used.

For peer review only

For peer review only - http://bmjopen.bmj.com/site/about/guidelines.xhtml

Introduction

A connection between clinicians and patients is central to clinical care [1]. Both, clinicians and patients have emotions, show emotions and will interpret each other's emotions [2, 3]. Despite their centrality, the clinicians' own emotions have largely remained unaddressed in medical education and practice [4], and have often been deemed as unprofessional [5-7], leaving clinicians with few skills to identify and manage their emotions. Within emotion management skills, a distinction can be made between 'other-directed' and 'self-directed' emotion skills. 'Other-directed' emotion skills are intended to improve the awareness, understanding and/or management of emotions/emotional states in others, while self-directed emotion skills are aimed to improve awareness, understanding and/or management of emotions/emotional states in the self, such as inward reflection or awareness of own emotions [8]. Since some emotion skills can be allocated to both (e.g. making soothing statements), a clearer distinction can be made according to the intended aim and focus of the emotion skill. To date, the majority of emotion awareness and emotion regulation training in medical education has been focused on emotions directed to the other, for example, emphasizing the development of empathy or compassion skills towards patients [8].

Nevertheless, some competency frameworks of reference for medical training include and expect some self-directed emotion awareness and other related skills. The CanMEDS framework was firstly developed in 1990 and serves as a base for many derived frameworks worldwide, foremost describing the competencies which physicians have to develop during their education [9]. In the CanMEDS, in their role as Professionals, physicians are expected to have an "applied capacity for self-regulation, including the assessment and monitoring of one's thoughts, behaviours, emotions, and attention for optimal performance and well-being", as well as to have a "mindful and reflective approach to practice", and "resilience for sustainable practice" [9]. For the training of medical students, Switzerland recently introduced the PROFILES framework, which is based on a combination of competencies adapted from the CanMEDS framework and Entrustable Professional Activities (EPAs) [10, 11]. Here, physician-led regulation and maintenance of the physician's own health, recognizing

For peer review only

excessive stress or recognizing personal illness, as well as being aware of own limits, and seeking supervision, are some of the expected skills that medical students should learn to display as professionals.

Despite these expectations from training, there seems to remain a gap in medical education in addressing and teaching physicians and medical students the skills to identify and manage their own emotions triggered by human interactions within the clinical care environment [12-15], and to recognize the impact they may have on clinical care or on the clinicians' wellbeing.

Significant high rates of poor mental health including burnout, emotional exhaustion, depression, anxiety, and suicidal ideation are well documented in medical students and in practicing physicians [16-19]. However, the number of preventative interventions and programs remain sparse and scattered in clinical practice and medical education. Some articles point out factors, such as chronically high workloads or lack of work-life balance which can help explain medical students' and physicians' decreased wellbeing [20, 21]. Other studies point to the important role that emotional blunting, numbness or frequent suppression of own emotions, due to the lack of emotion skills, have on the poor mental health of medical students and physicians [12-14, 22-24].

This scoping review will focus on identifying approaches to teaching medical students about the role of their own emotions in patient care along with the methods employed for self-directed emotion regulation training. To classify the different teaching methods, we will employ the structure developed by Mitchell et al. [25], which includes lectures, workshops, and other emotion skills training initiatives. For the development of future recommendations and interventions, it is necessary to obtain a comprehensive understanding of current teaching approaches to 'self-directed' emotion regulation training being implemented in medical education. For this purpose, a scoping review is justified and appropriate. A preliminary search of MEDLINE, the Cochrane Database of Systematic Reviews and *JBI Evidence Synthesis* was conducted and no current or underway systematic reviews or scoping reviews on the topic of medical students' management of own emotions or self-directed emotion skills training were identified. One related work identified has a focus on 'other-directed'

For peer review only

For peer review only - http://bmjopen.bmj.com/site/about/guidelines.xhtml

emotion skills training in medical education [8], and therefore the need for an equivalent work completing the picture of self-directed teaching approaches remains. This review will provide invaluable understandings that will facilitate the development of future training initiatives.

Objectives

The objective of this scoping review is to identify what approaches have been implemented in medical education programs to teach medical students the skills to identify and manage emotions that they may experience during physician-patient interactions and in the clinical environment in general.

Research questions

1. What specific teaching approaches, including lectures and training programs have been used in medical education to address the emotions physicians experience in the clinical environment?
2. With what teaching methods do specific lectures or related teaching formats cover elements related to physicians' 'self-directed' emotion regulation training during medical education?
3. At what stage during medical education do specific lectures or related teaching formats cover elements related to training physicians to recognize and regulate their own emotions?
4. Which specific aspects of physicians' own emotions, and which specific emotions, are addressed in 'self-directed' emotion regulation training initiatives in medical education?

Ethics and dissemination

This study will review existing literature on emotion awareness and emotion regulation training in medical education, and as a systematic scoping review does not require ethical approval. The results of this scoping review will be submitted for publication to relevant peer-reviewed journals and will be used to inform the development and implementation of training programs and research studies aimed at preparing medical students to identify and regulate their own emotions in the clinical environment.

For peer review only - http://bmjopen.bmj.com/site/about/guidelines.xhtml

Methods and analysis

Eligibility Criteria

The PCC (Participants, Concept, Context) eligibility criteria of the JBI framework has been used [26].

Patient and Public involvement

There is no direct patient or public engagement planned for this project. This is a systematic scoping review of the existing peer reviewed literature on the topic of interest. It is possible, however, that some of the literature included for data analyses might have had patient engagement or public involvement to some degree.

Participants/Population

Studies to be included must report on students during medical school training, excluding those who have obtained the professional degree to practice as physicians and who may have progressed to residency, specialty training, or who may have already started to practice as junior doctors. Studies reporting on students from other related fields (e.g. nursing, dentistry, or physiotherapy, etc.) are to be excluded.

Concept

Literature to be included reports on teaching elements of medical programs, workshops implemented, or related implementation formats of content aimed at addressing emotions physicians may experience during clinical encounters or in the clinical environment, as well as 'self-directed' emotion regulation training. Literature is to be excluded if only descriptive of mental health of medical students, only addressing needed changes on the topic of training of physicians' emotions without implementing teaching elements, or only reporting on related concepts without addressing elements

For peer review only

of physicians' self-directed emotion awareness and emotion regulation skills (e.g., other-directed emotion teaching, relaxation training, etc.).

Context

To identify teaching approaches, studies to be included must report on the medical teaching environment, either about online or campus-based programs, seminars, interventions, or related teaching formats. International studies from all countries and languages are to be included, no limit back to a certain year will be set to include the literature.

Types of evidence sources

In this review, we will include experimental and quasi-experimental study designs including randomized controlled trials, non-randomized controlled trials, and before and after studies. Exploratory study designs, such as qualitative or mixed method studies or related study designs will also be included. In addition, studies of any design describing the interventions but not the specific outcomes will also be included. We will exclude books, editorials, comments, posters, and opinion pieces.

Support

Two research librarians/medical information specialists of the department of the ISPM of the University of Bern supported the development of the search strategy with their expertise. Further consultations with the research librarians are planned throughout the review process.

Registration

This scoping review protocol is registered in Figshare (10.6084/m9.figshare.23642208). Any adjustments or modifications made throughout the analysis process will be documented and stated

For peer review only

in the final scoping review publication. An updated version of the scoping protocol will be available at Figshare.

Search strategy

This scoping review will be conducted in accordance with the JBI methodology for scoping reviews [26] and the Preferred Reporting Items for Systematic reviews and Meta-Analyses extension for Scoping Reviews (PRISMA-ScR) Checklist [27]. This scoping review protocol follows PRISMA-P guidelines (Moher et al. 2015) by employing the items recommended for scoping review protocols by Peters, Godfrey [26]. The protocol was developed using the template from the JBI Scoping Review Methodology Group [28].

After some initial meetings to define the focus of the research strategy and the keywords, the key concepts were chosen, and the final search strategy was peer reviewed with the librarians before it was run. The final key concepts and the search strategy for one database can be found in Table 1. For the literature search, a sensitive approach was chosen [29]. The following databases were searched: Medline (Ovid), PsychInfo (Ovid), ERIC (Ovid), CINAHL (EBSCOhost) and Web of Science – Social Sciences Citation Index SSCI (Clarivate, from inception to May 15st, 2023). No Language restrictions were applied. A total of 6726 articles were identified. The search strategy combined terms related to the population (medical students, trainees, or residents), exposure (medical education, teaching methods (undergraduate), curriculum) and outcomes (e.g. emotion regulation, psychological wellbeing, mental health). Despite our interest in medical students before obtaining the professional degree to practice as physicians, we included the terms “trainees” and “residents” in the search strategy to ensure that studies reporting on mixed populations were not excluded from the outset. In databases where a thesaurus was available, articles were searched by thesaurus terms, in other databases by free text-terms in title and abstract. Duplicate records were removed by using Deduklick [30], a fully automated deduplication algorithm. The full search strategies of all databases will be included in the appendix of the final scoping review publication.

For peer review only - http://bmjopen.bmj.com/site/about/guidelines.xhtml

Additionally, a grey literature search will be conducted, screening relevant literature references found through included full-texts and through hand searches. This search will include books and other sources such as MedEdPortal.org and similar resources. Experts in the field will also be asked for additional publications. An adapted PRISMA 2020 flow diagram describing the individual data sources and its output will be included in the scoping review publication [31].

Table 1: Key concepts for one full search strategy at Medline ALL (via Ovid)

**1) Medical education, teaching methods (undergraduates), curriculum**
(education, medical/ or education, medical, undergraduate/ or exp Curriculum/ or exp Educational Technology/ or (((medical or clinical OR hidden OR informal) adj2 (curricul* or training* or educat* or school or teaching or undergraduate*)) or Entrustable Professional Activit* or "Situations as Starting Point*" or "problem-based" or "project-based*" or ((teaching or training) adj3 (method* or type* or form* or strateg* or intervention* or technique* or setting*)) or academic training* or pedagog* or paedagog* or handbook* or "how to teach" or ((new or innovat* or classroom* or peer* or practice-based or bed-side or bedside or community or interactive* or creativ* or experimental* or precision or reflective) adj3 (teach*)) or "innovat* method*" or "diagnostic teaching" or ((small or large or discussion* or project*) adj1 group*) or "meet the expert*" or tutorial* or "hands on" or "psychomotor skills training*" or "case method*" or "case study method*" or "feedback session*" or debat* or quiz* or brainstorm* or lecture* or presentation* or "hot topic*" or webinar* or seminar* or symposi* or "case-based stud*" or "panel discussion*" or video* or movie* or film* or cinema* or "TED talk*" or "knowledge test*" or "facilitat* question* answer*" or "poster presentation*" or simulation* or "role-play*" or interview* or "patient simulat*" or "flipped classroom*" or forum* or game* or gaming or gamification or "skill demonstration*" or "skills demonstration*" or "mobile teaching app*" or "social media" or "scenario based" or "ward-round*" or "grand round*" or "practice exercise*" or "shadow expert*" or "inquiry-based" or "programmed instruct*" or "study assignment teach*" or "audiovisual instruction*" or "direct instruction*" or "individualized instruction*" or "individualised instruction*" or microteaching or "micro-teaching" or "multimedia instruction*" or scaffolding or "web based instruction*" or tutoring or workshop* or "virtual classroom*" or "group instruction*" or "formative assessment*" or debrief* or "information and communication technolog*").ab,ti,kf)
**2) Medical students**
(Students, Medical/ OR (((medical or healthcare or health-care or medicine) adj1 (student* or trainee* or resident*))).ab,ti)
**3) Emotion (regulation), psychological wellbeing, mental health**
(Emotions/ OR Mental Health/ OR (emotion or emotions or feelings or "emotional intelligen*" or emotionalit* or "emotional competenc*" or self-aware* or self-regulat* or (emotion* adj3 regulat*) or ((emotion* or intens*) adj2 reaction*) or "intens* feeling*" or self-reflect* or "reflective practice*" or self-care* or mindfulness or "self compassion*" or "coping strateg*" or psychological wellbeing or psychological well-being or mental health).ab,ti)
**4) Limits (no conference abstracts, letters, etc.)**
NOT (letter or news or comment or editorial or congress).pt.
**Translation of search strategy into Medline ALL (via Ovid)**
Segment PPEZ (Ovid MEDLINE® Epub Ahead of Print, In-Process & Other Non-Indexed Citations, Ovid MEDLINE® Daily, Ovid MEDLINE and Versions® 1946 to Present with Daily & Weekly Update)<br><br>(education, medical/ or education, medical, undergraduate/ or exp Curriculum/ or exp Educational Technology/ or (((medical or clinical OR hidden OR informal) adj2 (curricul* or training* or educat* or school or teaching or

For peer review only - http://bmjopen.bmj.com/site/about/guidelines.xhtml

undergraduate*)) or Entrustable Professional Activit* or "Situations as Starting Point*" or "problem-based" or "project-based*" or ((teaching or training) adj3 (method* or type* or form* or strateg* or intervention* or technique* or setting*)) or academic training* or pedagog* or paedagog* or handbook* or "how to teach" or ((new or innovat* or classroom* or peer* or practice-based or bed-side or bedside or community or interactive* or creativ* or experimental* or precision or reflective) adj3 (teach*)) or "innovat* method*" or "diagnostic teaching" or ((small or large or discussion* or project*) adj1 group*) or "meet the expert*" or tutorial* or "hands on" or "psychomotor skills training*" or "case method*" or "case study method*" or "feedback session*" or debat* or quiz* or brainstorm* or lecture* or presentation* or "hot topic*" or webinar* or seminar* or symposi* or "case-based stud*" or "panel discussion*" or video* or movie* or film* or cinema* or "TED talk*" or "knowledge test*" or "facilitat* question* answer*" or "poster presentation*" or simulation* or "role-play*" or interview* or "patient simulat*" or "flipped classroom*" or forum* or game* or gaming or gamification or "skill demonstration*" or "skills demonstration*" or "mobile teaching app*" or "social media" or "scenario based" or "ward-round*" or "grand round*" or "practice exercise*" or "shadow expert*" or "inquiry-based" or "programmed instruct*" or "study assignment teach*" or "audiovisual instruction*" or "direct instruction*" or "individualized instruction*" or "individualised instruction*" or microteaching or "micro-teaching" or "multimedia instruction*" or scaffolding or "web based instruction*" or tutoring or workshop* or "virtual classroom*" or "group instruction*" or "formative assessment*" or debrief* or "information and communication technolog*").ab,ti,kf) AND (Students, Medical/ or (((medical or healthcare or health-care or medicine) adj1 (student* or trainee* or resident*))).ab,ti) AND (Emotions/ or Mental Health/ or (emotion or emotions or feelings or "emotional intelligen*" or emotionalit* or "emotional competenc*" or self-aware* or self-regulat* or (emotion* adj3 regulat*) or ((emotion* or intens*) adj2 reaction*) or "intens* feeling*" or self-reflect* or "reflective practice*" or self-care* or mindfulness or "self compassion*" or "coping strateg*" or psychological wellbeing or psychological well-being or mental health).ab,ti) NOT (letter or news or comment or editorial or congress).pt

Data Extraction

For documenting and managing the search results, the Rayyan software will be used [32], as well as Endnote [33]. In a first step, identified literature will be screened by the first author and by a second reviewer independently at a title and abstract level. This will be followed by a full-text check of the literature that complied with the inclusion criteria during the first screening process. The full-text screening will also be performed by the two reviewers independently. For piloting purposes of the accuracy and sensitivity of the inclusion and exclusion criteria, 20% of the papers will be screened by the first and last author of this protocol. In case of disagreement at any of the screening phases, discussions will be held between the two screeners, and when needed, a third reviewer will be involved to reach an agreement.

Of the full texts selected in the second screening phase, data will be extracted for analysis. To chart the data, an extraction tool (Table 2) has been developed and will be piloted to test its feasibility. After the piloting, possible adaptations of the extraction tool will be made, and the final completed extraction tool will be published in the scoping review. As the studies to be included may contain interventions, the extraction tool includes a combination of applicable items from the TIDieR (Template for Intervention Description and Replication) and the PAGER (Patterns, Advances, Gaps,

For peer review only

Evidence for practice, Research recommendations) frameworks, as well as additional variables added by the authors. Data will be extracted using Covidence, a web-based software for systematic reviews, to allow for collaboration, tracking of changes, and secure storage. Once data extraction has been completed, the datasheet will be imported for analyses into MAXQDA [34].

Table 2: Extraction Tool. The rows of this table represent the columns of the extraction tool to be used.

Study characteristics / Patterns	*Study DOI*
	Title of publication
	Year of publication
	Name(s) of author(s)
	Email address corresponding author
	Aim(s) of the research study
	Country in which study was conducted
	Name Medical School / Institution where teaching was held
	Year(s) in which study was conducted
	Short description of year(s)/ period in which study was conducted
	Type of study design
	Emotions topic
Participants	*Healthcare area*
	Participant numbers total included (table)
	Age participant numbers total included (table)
	Participation numbers (completed intervention) (table)
	Age participation numbers (completed intervention) (table)
	Method of recruitment of participants (selection of: phone, Mail, email, medical clinic, during lecture/course, not reported, other)
TIDieR Framework and additional items added by the authors / Advances	*Aim of intervention*
	Brief name of intervention
	Main competencies addressed in the intervention / main competencies taught
	Specific context of intervention
	Procedures: each of procedures / activities and/or processes used in intervention (including enabling or support activities)
	Who provided the intervention
	Modes of delivery
	Delivery provided individually or in group? (selection of: group, individually, other)
	type(s) of location(s) for intervention (including infrastructure, etc.)

For peer review only - http://bmjopen.bmj.com/site/about/guidelines.xhtml

	Number of interventions
	Number of sessions intervention 1 was delivered
	Number of sessions intervention 2 was delivered
	Number of sessions intervention 3 was delivered
	Number of sessions intervention 4 was delivered
	Period of time intervention 1 was delivered
	Period of time intervention 2 was delivered
	Period of time intervention 3 was delivered
	Period of time intervention 4 was delivered
	Schedule
	Duration of session for Intervention 1
	Duration of session for Intervention 2
	Duration of session for Intervention 3
	Duration of session for Intervention 4
	Who received? (year of study, which students?) (open text field)
	Who received the intervention (selection box)<br>*(selection of: medical students during non-clinical years, medical students during clinical years, mixed medical students and postgraduates, mixed medical students and undergraduates (undergraduate: not at medical school yet), unclear, other)*
	Mandatory or elective training?<br>*(selection of: mandatory, elective, not reported, other)*
	Tailoring planned?<br>*(selection of: yes, no, other)*
	tailoring planned?
	If yes: what, why, when, how (for each modification)
	Modifications to intervention during course of study?<br>*(yes, no, other)*
	If yes (which modifications during course of study)<br>*(what, why, when, how / per for each modification)*
Outcomes	*Outcomes measured*<br>*(yes, no, other)*
	Outcomes assessed
	(Significant) Results / Conclusions / Outcomes
	Was program evaluated?<br>*(yes, no, other)*
Remaining part of PAGER framework that was not yet covered by the previous items	*Gaps / Research recommendations*
	Evidence for practice
	Limitations of the study (designs) reported by the author(s) / Challenges in training that may explain results
Source of funding	*Details source of funding*

Data Analyses and Presentation

For peer review only - http://bmjopen.bmj.com/site/about/guidelines.xhtml

The analysis of the data will be narrative and descriptive, presenting the results in forms of diagrams and tables and describing the literature included in terms of conceptual categories such as intervention type, duration of intervention, timing of the intervention across the curriculum, key findings, research gaps, as well as related categories relevant to the study questions.

Discussion

This scoping review will provide an overview of the approaches used in medical education to teach physicians on identifying and managing the emotions they may experience during physician-patient interactions or in the clinical environment, including 'self-directed' emotion regulation training. The review could help identify common themes, formats, and methods used to teach medical students about how to be aware of and how to manage their own emotions, the effectiveness of these initiatives, and identify gaps in the curriculum of medical students in that very field of training and education for their future as physicians. Ultimately, the results of this scoping review could provide recommendations to inform the future implementation of training programs in medical education to address physicians' own emotions in the clinical environment.

Declarations

Acknowledgements

The authors thank the librarian Beatrice Minder from the University Library of Bern for her support and help with designing the search strategies and Doris Kopp-Heim from the University Library of Bern for the peer-review of the searches using PRESS (Peer Review of Electronic Search Strategies: 2015 Guideline) [35]. The authors would also like to thank Lina Murer, master's student in Psychology, for supporting this project and for being the second independent screener of the literature screening process. This scoping review is to contribute towards the PhD degree of the first author (AL).

Funding

For peer review only

This scoping review is being funded by the the Schweizerischer Nationalfonds zur Förderung der Wissenschaftlichen Forschung (Swiss National Science Foundation (SNSF)) via an Eccellenza Professorial Fellowship grant to the last author (SCZ) (grant number PCEFP1_194177). The funder has had no role in the development of this protocol and will not be involved in any of the scientific steps required to complete the scoping review.

Author contributions

AL and SCZ conceived the idea of the study and developed the initial design of the scoping review. AL, RB, SGS, and SCZ contributed to the methodological design of the review. AL and SCZ drafted the manuscript. All authors critically revised the manuscript for important intellectual content. SCZ is the guarantor of the systematic scoping review.

Conflicts of interest disclosures

The authors do not have any conflicts of interest to report.

For peer review only

For peer review only - http://bmjopen.bmj.com/site/about/guidelines.xhtml

References

1. Brown-Johnson, C., et al., *What is clinician presence? A qualitative interview study comparing physician and non-physician insights about practices of human connection.* BMJ Open, 2019. **9**(11): p. e030831.
2. Schwartz, R., J.A. Hall, and L.G. Osterberg, *Emotion in the Clinical Encounter*. 2021: McGraw-Hill Education.
3. Roter, D.L., et al., *The Expression of Emotion Through Nonverbal Behavior in Medical Visits.* Journal of General Internal Medicine, 2006. **21**(S1): p. S28-S34.
4. Holt Siropaides, C., M. Howell, and C.L. Chou, *Teaching about Emotions in Healthcare*, in *Emotion in the Clinical Encounter*, R. Schwartz, J.A. Hall, and L.G. Osterberg, Editors. 2021, McGraw Hill. p. 1-9.
5. Sung, A.D., et al., *Crying: experiences and attitudes of third-year medical students and interns.* Teaching & Learning in Medicine, 2009. **21**(3): p. 180-187.
6. Zambrano, S.C., A. Chur-Hansen, and B. Crawford, *On the emotional connection of medical specialists dealing with death and dying: a qualitative study of oncologists, surgeons, intensive care specialists and palliative medicine specialists.* BMJ Supportive & Palliative Care, 2012. **2**(3): p. 270-275.
7. Kerasidou, A. and R. Horn, *Making space for empathy: supporting doctors in the emotional labour of clinical care.* BMC Medical Ethics, 2016. **17**(1): p. 8.
8. Satterfield, J.M. and E. Hughes, *Emotion skills training for medical students: a systematic review.* Medical Education, 2007. **41**(10): p. 935-941.
9. Royal College of Physicians and Surgeons of Canada. *CanMEDS Framework*. n.d. [cited 2023 19.06.2023]; Available from: www.royalcollege.ca/rcsite/canmeds/canmeds-framework-e.
10. Swiss Working Group for PROFILES Implementation (SWGPI). *PROFILES*. n.d. [cited 2023 19.06.2023]; Available from: www.profilesmed.ch.
11. Sohrmann, M., et al., *Nationwide introduction of a new competency framework for undergraduate medical curricula: a collaborative approach.* Swiss Med Wkly, 2020. **150**: p. w20201.
12. Doulougeri, K., E. Panagopoulou, and A. Montgomery, *(How) do medical students regulate their emotions?* BMC Medical Education, 2016. **16**(1).
13. Linda Hadorn, B.W., Carlos B. Quinto, *Nachholbedarf in Sachen Ärztegesundheit.* SCHWEIZERISCHE ÄRZTEZEITUNG, 2020. **38**(101): p. 1171-1172.
14. Shapiro, J., *Perspective: Does medical education promote professional alexithymia? A call for attending to the emotions of patients and self in medical training.* Acad Med, 2011. **86**(3): p. 326-32.
15. Ungar, P., et al., *Online programs to strengthen the mental health of medical students: A systematic review of the literature.* Medical Education Online, 2022. **27**(1): p. 2082909.
16. Almutairi, H., et al., *Prevalence of burnout in medical students: A systematic review and meta-analysis.* International Journal of Social Psychiatry, 2022. **68**(6): p. 1157-1170.
17. Devi, S., *Doctors in distress.* The Lancet, 2011. **377**(9764): p. 454-455.
18. Tugnoli, S., et al., *Parental bonding, depression, and suicidal ideation in medical students.* Frontiers in Psychology, 2022. **13**: p. 877306.
19. Ernst, J., et al., *Burnout, depression and anxiety among Swiss medical students – A network analysis.* Journal of Psychiatric Research, 2021. **143**: p. 196-201.

For peer review only - http://bmjopen.bmj.com/site/about/guidelines.xhtml

20. West, C.P., L.N. Dyrbye, and T.D. Shanafelt, *Physician burnout: contributors, consequences and solutions.* Journal of Internal Medicine, 2018. **283**(6): p. 516-529.
21. IsHak, W., et al., *Burnout in medical students: a systematic review.* 2013: p. 242–245.
22. Ardenghi, S., et al., *The role of difficulties in emotion regulation in predicting empathy and patient-centeredness in pre-clinical medical students: a cross-sectional study.* Psychology, Health & Medicine, 2021: p. 1-15.
23. Shapiro, J., *The feeling physician: educating the emotions in medical training.* European Journal for Person Centered Healthcare, 2012. **1**(2): p. 310-316.
24. Dobkin, P.L. and T.A. Hutchinson, *Teaching mindfulness in medical school: where are we now and where are we going?* Med Educ, 2013. **47**(8): p. 768-79.
25. Mitchell, S., et al., *Taxonomy of teaching methods and their use in health professions education: a scoping review protocol.* BMJ Open, 2024. **14**(1): p. e077282.
26. Peters, M., et al., *Best practice guidance and reporting items for the development of scoping review protocols.* JBI evidence synthesis, 2022. **Publish Ahead of Print**.
27. Tricco, A.C., et al., *PRISMA Extension for Scoping Reviews (PRISMA-ScR): Checklist and Explanation.* Annals of Internal Medicine, 2018. **169**(7): p. 467-473.
28. JBI Scoping Review Methodology Group. *JBI*. n.d. [cited 2023 03.10.2023]; Available from: www.jbi.global/scoping-review-network/resources.
29. Peters, M.D.J., et al., *Updated methodological guidance for the conduct of scoping reviews.* JBI Evidence Synthesis, 2020. **18**(10): p. 2119-2126.
30. Borissov, N., et al., *Reducing systematic review burden using Deduklick: a novel, automated, reliable, and explainable deduplication algorithm to foster medical research.* Systematic Reviews, 2022. **11**(1): p. 172.
31. Page, M.J., et al., *The PRISMA 2020 statement: an updated guideline for reporting systematic reviews.* BMJ, 2021: p. n71.
32. Ouzzani, M., et al., *Rayyan—a web and mobile app for systematic reviews.* Systematic Reviews, 2016. **5**(1).
33. The EndNote Team, *EndNote*. 2013, Clarivate: Philadelphia, PA.
34. VERBI Software, *MAXQDA 2024*. 2021, VERBI Software: Berlin, Germany.
35. Mcgowan, J., et al., *PRESS Peer Review of Electronic Search Strategies: 2015 Guideline Statement.* Journal of Clinical Epidemiology, 2016. **75**: p. 40-46.

For peer review only

Physicians' emotions and emotion skills training during medical education: a systematic scoping review protocol
Lange et al., submitted 2023

Recommended items to report in a scoping review protocol (JBI Scoping Review Methodology Group, n.d.; Peters et al., 2022)

Section and topic	Item No	Checklist item	Page#
ADMINISTRATIVE INFORMATION			
Title: *Physicians' emotions and emotion skills training during medical education: a systematic scoping review protocol*			
Identification	1a	Identify the report as a protocol of a scoping review	1
Update	1b	If the protocol is for an update of a previous scoping review, identify as such	n.a.
Registration	2	If registered, provide the name of the registry (such as JBI) and registration number	8
Authors:			
Contact	3a	Provide name, institutional affiliation, email address of all protocol authors; provide physical mailing address of corresponding author	1
Contributions	3b	Describe contributions of protocol authors and identify the guarantor of the review	
Amendments	4	If the protocol represents an amendment of a previously completed or published protocol, identify as such and list changes; otherwise, state plan for documenting important protocol amendments	8
Support:			
Sources	5a	Indicate sources of financial or other support for the review	8, 14
Sponsor	5b	Provide name for the review funder and/or sponsor	14
Role of sponsor or funder	5c	Describe roles of funder(s), sponsor(s), and/or institution(s), if any, in developing the protocol	14
INTRODUCTION			
Rationale	6	Describe the rationale for the review in the context of what is already known (Note: Consider providing a rationale for the choice of conducting a scoping review as compared to other evidence synthesis approaches)	4 - 6
Objectives	7	Provide an explicit statement of the question(s) the review will address with reference to the inclusion/ exclusion criteria	6
METHODS			
Eligibility criteria	8	Specify the study characteristics (such as PICO, study design, setting, timeframe) and report characteristics (such as years considered, language, publication status) to be used as criteria for eligibility for the review	7 - 8
Information sources	9	Describe all intended information sources (such as electronic databases, contact with study authors, trial registers or other gray literature sources) with planned dates of coverage	8 - 9
Search strategy	10	Present draft of search strategy to be used for at least one electronic database, including planned limits, such that it could be repeated	9 - 10
Study records:			
Data management	11a	Describe the mechanism(s) that will be used to manage records and data throughout the review	11
Selection process	11b	State the process that will be used for selecting studies (such as two independent reviewers) through each phase of the review (that is, screening, eligibility and inclusion)	7 - 8
Data collection process	11c	Describe planned method of extracting data from reports (such as piloting forms, done independently, in duplicate), any processes for obtaining and confirming data from investigators	10 - 12
Data items	12	List and define all variables for which data will be sought (such as PICO items, funding sources), any preplanned data assumptions and simplifications (Note: Scoping reviews may not use PICO and instead may use JBI's Population, Concept, and Context [PCC] or another approach to reporting eligibility criteria)	11 - 12

For peer review only

Physicians' emotions and emotion skills training during medical education: a systematic scoping review protocol Lange et al., submitted 2023

Outcomes and prioritization	13	List and define all outcomes for which data will be sought, including prioritization of main and additional outcomes, with rationale (Note: Scoping reviews may not extract outcome data, so this can refer to whichever data items are extracted)	11-12
Risk of bias in individual studies	14	If this is to occur, describe anticipated methods for assessing risk of bias of individual studies, including whether this will be done at the outcome or study level, or both; state how this information will be used in data synthesis (Note: Scoping reviews typically do not include risk of bias assessment, but this information should be described if it will occur)	n.a.
Data synthesis	15a	Describe criteria under which study data will be presented (Note: Scoping reviews do not typically include quantitative synthesis of study data, but should still describe in advance how extracted data are anticipated to be presented in the resulting review)	12
	15b	Describe the planned approach to how extracted data will be presented (such as figures, tables, evidence gaps maps)	12
	15c	Describe any proposed additional analyses (such as thematic analyses) (Note: The JBI methodological guidance does not recommend undertaking thematic analysis as this synthesis of data should ideally occur following methodological appraisal of the included sources)	12
	15d	If quantitative synthesis is not appropriate, describe the type of summary planned	12
Meta-bias(es)	16	Specify any planned assessment of meta-bias(es) (such as publication bias across studies, selective reporting within studies) (Note: Scoping reviews typically do not include assessment of meta-bias[es], but this information should be described if it will occur)	n.a.
Confidence in cumulative evidence	17	Describe how the strength of the body of evidence will be assessed (such as GRADE)(GRADE for scoping reviews currently does not exist, and at this stage it is unclear if a variation on GRADE would be useful)	n.a.

References

JBI Scoping Review Methodology Group. (n.d.). *JBI*. Retrieved 03.10.2023 from www.jbi.global/scoping-review-network/resources

Peters, M., Godfrey, C., McInerney, P., Khalil, H., Larsen, P., Marnie, C., Pollock, D., Tricco, A., & Munn, Z. (2022). Best practice guidance and reporting items for the development of scoping review protocols. *JBI Evidence Synthesis*, *Publish Ahead of Print*. https://doi.org/10.11124/JBIES-21-00242

For peer review only
